# Supplementary material for: Perspectives of compounding pharmacists on alcohol-based hand sanitizer production and utilization for COVID-19 prevention in Addis Ababa, Ethiopia: A descriptive phenomenology study
Source: PLoS One. 2021 Apr 29;16(4):e0250020. doi: 10.1371/journal.pone.0250020 (PMC8084187; doi:10.1371/journal.pone.0250020)
Supplement: S1 File — (DOCX) [file pone.0250020.s001.docx]

# Annexes

## Annex 1. Information sheet

Attached herewith is a key informant guide developed explore the experience and views of compounding pharmacists in public hospitals about production and utilization of hospital-based ABHR solution in Addis Ababa, Ethiopia. This study is conducted by MOH, PMED.

Your participation is purely voluntary and information you provide will be kept completely confidential. Your name will never be written and aggregate responses from different respondents are identified only by codes. However, tape recorder will be used during the interview.

Your honest response to the questions is of paramount importance for the successful completion of the study. There is no Right or Wrong answer and you can have clarification for any doubt regarding the questions.

.

Are you willing to respond to the questions? Yes 🞏 No 🞏

## Annex 2. Key-informant Interview Guide

**Part one: Socio-demographic characteristic of key informant**

**Direction**: please put /**🗸**/ mark in the box provided or write your response on space provided.

Date of interview: ______/________/_______

**Part I: Key informant characteristics**

| 1 | Age (years) | ______________ |
| --- | --- | --- |
| 2 | Gender | 🖵Male 🖵Female |
| 3 | Highest Qualification | 🖵 MSc 🖵BPharm 🖵Druggist  🖵 Other Specify: _______________ |
| 4 | Current practice setting | 🖵 Specialized Hospital  🖵 General Hospital  🖵 Other Specify, _______________ |
| 5 | Present position | ________________________________ |
| 6 | Year of experience, total (years) | ________________________________ |
| 7 | Years of working experience in the current position (years) | ________________________________ |
| 8 | When did your facility start production (mm/yy)? | _________/____________ |

**Part two: key-informant guides**

1. **Nature of formulation**
2. Does your facility follow World Health organization’s guideline for production of ABHR?

[*Probe: If “No”, ask formulation ingredients and amount to be used during production]*

1. **Availability and supply of chemicals, materials, PPE**
2. Is there problem in the supply of ingredients, personnel protective equipment, packaging and labeling materials? *[probe: reasons of unavailability: cost or procurement difficulties]*
3. What is the source of your active ingredient (ethanol or isopropyl alcohol), other chemicals and packaging and labeling materials?

*[Probe: sugar industry, chemical industry, purchase from whole sale, obtained locally, imported, cost of 100 ml dispenser (for comparison)]*

1. Do the required equipment specifically for the production of ABHRs purchased or donated or had them already?
2. **Standard of practice**
3. Do you think that the production of ABHR solution at your facility comply with the requirement or follow GCP principles?

*[Probe: regular calibration and/or qualification, relevant quality control activities ABHR production (alcohol content determination, hydrogen peroxide titrimetry)]*

1. How do you compare the ABHR solution prepared at your facility with those obtained from market? Do you have preference? What are the major reasons for your response?

*(Probe: Differences in effectiveness, quality, cost (100 ml of facility produced and purchased from market), manufacturing procedures, regulation, type of ingredients)*

1. **Production capacity and future plan**
2. Do you think the manufactured ABHR solution at your facility satisfy the demand of your organization? Explain the reasons for the response? Do you estimate/assess the demand (daily or weekly) of ABHR solution at your faculty?

Probe: production capacity (daily/weekly/monthly), barriers for adequate production]

1. Do your organization support/sell the product to other institutions/general public?

*(Probe: agreement or contacts, the market demand)*

1. Does your organization have a plan to continue ABHR solution production after the COVID-19 pandemic is controlled globally?
2. **Rational use of ABHR**
3. Is there proper use of dispenser bottles by the health care professionals?

(Probe: bring them properly for refill, request new if stolen or damage of pump or cup,…)

1. Do you think there is rational use of ABHR solution at the facility? Mention reasons for your response?
2. Do you consider yourself to serve as highly accessible information expert and provide evidence-based information and education about sanitizer (ABHR)? If so, what type of information did you provide so far?
3. **Professional**
4. Who is involved in production in your unit?

*[Probe: pharmacists, nurses, chemists]*

1. Do they take training? If so, who is organize the training and when?
2. Do you believe that pharmacy professionals are duly recognized and reimbursed for their public health services to combat COVID-19 in Ethiopia? If not, why?
3. What supports are required to reimburse pharmacy professional for their service related with COVID-19 epidemic?

*[Probe: accessing sanitizers, affordable price, advice in rational use, fair reimbursements]*

1. **Others**
2. What best practices does your institution have in ABHR production and utilization to prevent infections (COVID-19) epidemic?
3. Do you think your facility needs to be supported/strengthened/ with respect to ABHR solution production?

If so, who do you think should support it? How do you think should it be supported?

*[Probe: training, material/resource]*

If you have further points or comments to add, we will be appreciating.

**[Thank you!!!]**
