# Supplementary material for: Perspectives of compounding pharmacists on alcohol-based hand sanitizer production and utilization for COVID-19 prevention in Addis Ababa, Ethiopia: A descriptive phenomenology study
Source: PLoS One. 2021 Apr 29;16(4):e0250020. doi: 10.1371/journal.pone.0250020 (PMC8084187; doi:10.1371/journal.pone.0250020)
Supplement: S2 File — (DOCX) [file pone.0250020.s002.docx]

| **Topic** | | **Item No.** | | | **Guide Questions/Description** | **Answers** | **Reported on Page no.** |
| --- | --- | --- | --- | --- | --- | --- | --- |
| **Domain 1: Research team and reflexivity** | | | | | | | |
| *Personal characteristics* | | | | | | | |
| Interviewer/facilitator | 1 | | | Which author/s conducted the interview? | | AM, MN, MW | 7 |
| Credentials | 2 | | | What were the researcher’s credentials? E.g. PhD, MD | | MSc and PhD candidate | - |
| Occupation | 3 | | | What was their occupation at the time of the study? | | Pharmacy, Directorate, Lecturer, Assistant professor | - |
| Gender | 4 | | | Was the researcher male or female? | | Male | - |
| Experience and training | 5 | | | What experience or training did the researcher have? | | AM took advanced qualitative research methods course | - |
| *Relationship with participants* | | | | | | | |
| Relationship established | 6 | | | Was a relationship established prior to study commencement? | | Before starting the interviews, a friendly relationship was established with the consented participants | - |
| Participant knowledge of the interviewer | 7 | | | What did the participants know about the researcher? e.g. personal goals, reasons for doing the research | | Reasons for doing the research in-order to improve future ABHR production and utilization | 5 |
| Interviewer characteristics | 8 | | | What characteristics were reported about the inter viewer/facilitator? e.g. Bias, assumptions, reasons and interests in the research topic | | Reasons and interest in research topic | 7 |
| **Domain 2: Study design** |  | | | | | | |
| *Theoretical framework* | | | | | |  |  |
| Methodological orientation and Theory | 9 | | | What methodological orientation was stated to underpin the study? e.g. grounded theory, discourse analysis, ethnography, phenomenology, content analysis | | Descriptive phenomenology | 5 |
| *Participant selection* | | | | | | | |
| Sampling | 10 | | | How were participants selected? e.g. purposive, convenience, consecutive, snowball | | Purposeful sampling technique was applied to recruit the participants | 6 |
| Method of approach | 11 | | | How were participants approached? e.g. face-to-face, telephone, mail, email | | Face-to-face interviews | 7 |
| Sample size | 12 | | | How many participants were in the study? | | 13 pharmacy professionals | 6 |
| Non-participation | 13 | | | How many people refused to participate or dropped out? Reasons? | | No drop out | - |
| *Setting* | | | | | | | |
| Setting of data collection | 14 | | Where was the data collected? e.g. home, clinic, workplace | | | Workplace in compounding pharmacist’s office (Hospital) | 7 |
| Presence of non-participants | 15 | | Was anyone else present besides the participants and researchers? | | | Research assistant to take back-up notes | 7 |
| Description of sample | 16 | | What are the important characteristics of the sample? e.g. demographic data, date | | | Pharmacists (9 male, 4 female; 10 first degree, 3 second degree) | 9, Table 1 |
| *Data collection* | | | | | | | |
| Interview guide | 17 | | Were questions, prompts, guides provided by the authors? Was it pilot tested? | | | Yes, prompts were provided by the researchers. Piloting was done. | 7, 8 |
| Repeat interviews | 18 | | Were repeat interviews carried out? If yes, how many? | | | There were no repeat interviews. However, if case further clarifications were required from the consented participants, they were contacted through a phone call | - |
| Audio/visual recording | 19 | | Did the research use audio or visual recording to collect the data? | | | Audio recording | 7 |
| Field notes | 20 | | Were field notes made during and/or after the interview or focus group? | | | Yes | 7 |
| Duration | 21 | | What was the duration of the interviews? | | | 30 – 45 minutes | 7 |
| Data saturation | 22 | | Was data saturation discussed? | | | Yes | 7 |
| Transcripts returned | 23 | | Were transcripts returned to participants for comment and/or correction? | | | No, as there was no demand for it | - |
| **Domain 3: analysis and findings** | | | | | | | |
| *Data analysis* | | | | | | | |
| Number of data coders | 24 | | How many data coders coded the data? | | | 3 | - |
| Description of the coding tree | 25 | | Did authors provide a description of the coding tree? | | | Yes | 7 |
| Derivation of themes | 26 | | Were themes identified in advance or derived from the data? | | | Themes, subthemes and categories were derived from the data | 7 |
| Software | 27 | | What software, if applicable, was used to manage the data? | | | None | - |
| Participant checking | 28 | | Did participants provide feedback on the findings? | | | They were not contacted | - |
| *Reporting* | | | | | | | |
| Quotations presented | 29 | | Were participant quotations presented to illustrate the themes/findings? Was each quotation identified? e.g. participant number | | | Yes | 8-19 |
| Data and findings consistent | 30 | | Was there consistency between the data presented and the findings? | | | Yes | 9-19 |
| Clarity of major themes | 31 | | Were major themes clearly presented in the findings? | | | Yes | 9-19 |
| Clarity of minor themes | 32 | | Is there a description of diverse cases or discussion of minor themes? | | | Both | 9-24 |
