# Supplementary material for: Perspectives of compounding pharmacists on alcohol-based hand sanitizer production and utilization for COVID-19 prevention in Addis Ababa, Ethiopia: A descriptive phenomenology study
Source: PLoS One. 2021 Apr 29;16(4):e0250020. doi: 10.1371/journal.pone.0250020 (PMC8084187; doi:10.1371/journal.pone.0250020)
Supplement: S3 File — (DOCX) [file pone.0250020.s003.docx]

**Participant #01**

| Age | 35 |
| --- | --- |
| Gender | Male |
| Highest qualification | MSC in pharmaceutics |
| Current practices setting | Specialized hospitals |
| Present position | Compounding case team head |
| Year of experiences, total(year) | 12 |
| Year of working experiences in the current position (years) | 2 |
| When did your facility start production? | Before COVID-19 Pandemic |

1. **Nature of formulation**
2. Do you follow World Health organization’s guideline for production of ABHR? [*Probe: If “No”, ask formulation ingredients and amount to be used during production]*

Response: Yes, we are following WHO guidelines formulation 1 Ethanol based API.

1. **Availability and supply of chemicals, materials, PPE**
2. Is there problem in the supply of ingredients, personnel protective equipment, packaging and labelling materials? *[probe: reasons of unavailability: cost or procurement difficulties]*

Response: More or less we have the listed supplies but the major problem is consistent supply of ethanol. For our hospital consumption we have ethanol. However, based our production capacity we are not getting ethanol for others. The second big problem is packaging materials dispensing bottles. Due to this we are distributing pocket size only. Face mask also big problem as an institution. Show cover and head cover is not available. The reason is beyond the institutions but the first reason is lack of concentration by responsible body and unable to prioritized and PPE is not available at the market and there is lack of attention for the ABHR production.)

1. What is the source of your active ingredient (ethanol or isopropyl alcohol), other chemicals and packaging and labelling materials? *[Probe: sugar industry, chemical industry, purchase from whole sale, obtained locally, imported, cost of 100 ml dispenser (for comparison)]*

Response: Ethanol from sugar factory via FMOH and industry bureau from Metehara sugar factory. Bottles and labelling from local manufactures.

1. Do the required equipment specifically for the production of ABHRs purchased or donated or had them already?

Response: PPE from hospitals. our hospitals are working jointly with AAU pharmacy departments thus all the necessary equipment’s were available before the production of ABHR for educational lab and there is no special order.

1. **Standard of practice**
2. Do you think that the production of ABHR solution at your facility comply the requirement or follow GCP principles? *[Probe: regular calibration and/or qualification, relevant quality control activities ABHR production (alcohol content determination, H_2_0_2_ titrimetry)]*

Response: Yes. The hospitals are closely working with AAU and closely GCP are known. However, we are not fully applying GCP because we are not fulfilling different thing with regard to GCP but the core GCP like quality, sanitation and hygiene, precaution measure, bottle recycling process, documentation is gaps. Instruments are calibrated like alcoholometers and distilled water sample and hydrogen peroxide titration is worked.

1. How do you compare the ABHR solution prepared at your facility with those obtained from market? Do you have preference? Why? *(Probe: Differences in effectiveness, quality, cost (100 ml of facility produced and purchased from market)*

Response: All institutions quality should be evaluated by other third body organ. Quality of products and decoration is one type of quality parameters and we are definitely assuring quality of products. Based on this EFDA gives production licencing.

1. If you think there is difference in any of the attributes, what are the major reasons? *(Probe: manufacturing procedures, regulation, type of ingredients etc.)*

Response: All institutions quality should be evaluated by other third body organ. Quality of products and decoration is one type of quality parameters and we are definitely assuring quality of products. Based on this EFDA gives production licencing

1. **Production capacity and future plan**
2. Do you think the manufactured ABHR solution at your facility satisfy the demand of your organization? Explain the reasons for the response? Probe: production capacity (daily/weekly/monthly), barriers for adequate production]

Response: We are starting this production before COVID -19 pandemic but we are produced the products consistently on February and our hospital demand is 830 litters per week and currently with our any problem we are producing as per the hospital demand but as community level we are not satisfying the demand because of enough supply of ethanol and packaging.

1. Do your organization support/sell the product to other institutions/general public? *(Probe: agreement or contacts, the market demand)*

Response: Yes, we gave our products for FMOH AAU Community and health centres via official letters but there are no formal agreements.

1. Does your organization have a plan to continue ABHR solution production after the COVID-19 pandemic is controlled globally?

Response: Yes, we have a plan to produces at the end of COVID-19 Pandemic because we were start the production before the pandemic and we made semi-automated machine and can produce in bulk

1. Do you estimate/assess the demand (daily or weekly) of ABHR solution at your faculty? If yes, how? If no, why?

Response: Yes, we know our hospital demand and the demand is 830 litters per a week. There is a gap on the estimation of the demand. we assigned the demand roughly, not based on study.

We produce 830 litters per two days.

1. **Rational use of ABHR**
2. Is there proper use of dispenser bottles by the health care professionals?

*(Probe: bring them properly for refill, request new if stolen or damage of pump or cup)*

Response: No. there is no proper use of dispensing bottles

1. Do you think there is rational use of ABHR solution at the facility? Mention reasons for your response?

Response: No. there is no rational use because our dispatching in bulk for departments. but it needs further investigation

1. Do you consider yourself to serve as highly accessible information expert and provide evidence-based information and education about sanitizer (ABHR)? If so, what type of information did you provide so far?

Response: Yes. We gave inaugurate the small-scale unit for hospital higher officials and give presentation for them on the over view of ABHR specially on when how to use and part of essential medicine.

1. **Professional**
2. Who is involved in production in your unit? *[Probe: pharmacists, nurses, chemists]*

Response: We have 5 dedicated pharmacists in different team

1. Do they take training? If so, who is organize the training and when?

Response: Yes, training is facilitated by FMOH before 4 months. But, the rest of my colleagues are not trained but informally the teach

1. Do you believe that pharmacy professionals are duly recognized and reimbursed for their public health services to combat COVID-19 in Ethiopia? If not, why?

Response: The activities are not getting attentions from government. The main reason is the pharmacist are unable to surface out on the community and media.

1. What supports are required to reimburse pharmacy professional for their service related with COVID-19 epidemic? *[Probe: accessing sanitizers, affordable price, advice in rational use, fair reimbursements]*

Response: Institutionally there must be payment or incentives and the FMOH should convinces the hospital official on the HEALTH hazardous parts of ABHR production.

1. **Best practice**
2. What best practices does your institution have in ABHR production and utilization to prevent infections (COVID-19) epidemic?

Response: no response,

1. Do you think your facility needs to be supported/strengthened/ with respect to ABHR solution production? If so, who do you think should support it? How do you think should it be supported?

*[Probe: training, material/resource]*

Response: Recognition should be given by hospital officials and FMOH and training, quality types of activities should be done by professionals. FMOH should have convincing support of suppliers for hospitals.

**If you have further points or comments to add, we will be appreciating.**

All stake holders should work on the consistent supply of ABHR production. Secondly, we have to work strongly on the quality of products specially on self-inspection of pre and post production quality, other should not inspect our products and give warranty for our user.

**[Thank you!]**

**Participant #02**

| Age | 31 |
| --- | --- |
| Gender | Female |
| Highest qualification | BPharm |
| Current practices setting | General Hospital |
| Present position | Compounding team leader |
| Year of experiences, total(year) | 4 years |
| Year of working experiences in the current position (years) | 6 months |
| When did your facility start production? | After COVID-19 Pandemic |

1. **Nature of formulation**
2. Do you follow World Health organization’s guideline for production of ABHR? [*Probe: If “No”, ask formulation ingredients and amount to be used during production]*

Response: Yes, we are using WHO guidelines and additionally we prepare SOP based on WHO guidelines.

1. **Availability and supply of chemicals, materials, PPE**
2. Is there problem in the supply of ingredients, personnel protective equipment, packaging and labelling materials? *[probe: reasons of unavailability: cost or procurement difficulties]*

Response: there was scarcity of ethanol 96 % and alcoholometry.

1. What is the source of your active ingredient (ethanol or isopropyl alcohol), other chemicals and packaging and labelling materials? *[Probe: sugar industry, chemical industry, purchase from whole sale, obtained locally, imported, cost of 100 ml dispenser (for comparison)]*

Response: Ethanol direct procurement from whole sealers from universal and METHERA sugar factory via EFMO. Packaging materials simple printing.

1. Do the required equipment specifically for the production of ABHRs purchased or donated or had them already?

Response: Ethanol is purchased only for ABHR whereas other ingredients are already available. PPE materials are already available in our institutions.

1. **Standard of practice**
2. Do you think that the production of ABHR solution at your facility comply the requirement or follow GCP principles? *[Probe: regular calibration and/or qualification, relevant quality control activities ABHR production (alcohol content determination, H_2_0_2_ titrimetry)]*

Response: More or less we are following GCP.

1. How do you compare the ABHR solution prepared at your facility with those obtained from market? Do you have preference? Why? *(Probe: Differences in effectiveness, quality, cost (100 ml of facility produced and purchased from market)*

Response: In terms of quality, we are using ingredients as per WHO so this is the preferences

1. If you think there is difference in any of the attributes, what are the major reasons? *(Probe: manufacturing procedures, regulation, type of ingredients etc.)*

Response: The major deference is the ingredient that we use is as per WHO standard

1. **Production capacity and future plan**
2. Do you think the manufactured ABHR solution at your facility satisfy the demand of your organization? Explain the reasons for the response? Probe: production capacity (daily/weekly/monthly), barriers for adequate production]

Response: Yes, we are satisfying the facility demand but there was inconsistency supply of production due to scarcity of ethanol and now AAHRB gives ready-made sanitizers and now we consistently distribute it.

1. Do your organization support/sell the product to other institutions/general public? *(Probe: agreement or contacts, the market demand)*

Response: No, but we were putting as a plan. The reason is the procured ethanol was lost due to the container breakage and the cost of ethanol was very expensive. Thus, we were not distributed for market / community.

1. Does your organization have a plan to continue ABHR solution production after the COVID-19 pandemic is controlled globally?

Response: Yes, because it is a part of IP practices. if there is continues supply of ingredients, we have plan to produces

1. Do you estimate/assess the demand (daily or weekly) of ABHR solution at your faculty? If yes, how? If no, why?

Response: Rough estimation of 100 ml/week for public

1. **Rational use of ABHR**
2. Is there proper use of dispenser bottles by the health care professionals?

*(Probe: bring them properly for refill, request new if stolen or damage of pump or cup)*

Response: Yes, they have information is deaminated via media and others. Thus, there is proper use

1. Do you think there is rational use of ABHR solution at the facility? Mention reasons for your response?

Response: Yes, DIC and compounding unit gives information on rational use of ABHR

1. Do you consider yourself to serve as highly accessible information expert and provide evidence-based information and education about sanitizer (ABHR)? If so, what type of information did you provide so far?

Response: information on rational use of ABHR.

1. **Professional**
2. Who is involved in production in your unit? *[Probe: pharmacists, nurses, chemists]*

Response: Pharmacist and compounding focal person

1. Do they take training? If so, who is organize the training and when?

Response: No. Through informal way of training like reading and watching video on ABHR production we are preparing the product

1. Do you believe that pharmacy professionals are duly recognized and reimbursed for their public health services to combat COVID-19 in Ethiopia? If not, why?

Response: No. But in our hospital, we get a means of compensation as a duty for pharmacist. but there is no extra payment

1. What supports are required to reimburse pharmacy professional for their service related with COVID-19 epidemic? *[Probe: accessing sanitizers, affordable price, advice in rational use, fair reimbursements]*

Response: Training on ABHR production, Supply of raw materials Supply and building of production extra Room/premises

1. **Best practice**
2. What best practices does your institution have in ABHR production and utilization to prevent infections (COVID-19) epidemic?

Response: We were prepared production of ABHR without related training based on our SOP can be considered as best practices.,

1. Do you think your facility needs to be supported/strengthened/ with respect to ABHR solution production? If so, who do you think should support it? How do you think should it be supported?

*[Probe: training, material/resource]*

Response: Based the previous assessment gap, it is better to fulfilling the necessary ingredients, giving Training, material supply, alcoholmeters supply.

**If you have further points or comments to add, we will be appreciating.**

no.

**Participant #03**

|  | Age (years) | 28 |
| --- | --- | --- |
| 2 | Gender | Male |
| 3 | Highest Qualification | BPharm |
| 4 | Current practice setting | Specialized Hospital |
| 5 | Present position | Compounding coordinator |
| 6 | Year of experience, total (years) | 5 |
| 7 | Years of working experience in the current position (years) | 2 months |
| 8 | When did your facility start production? | After COVID-19 Pandemic |

1. **Nature of formulation**
2. Do you follow World Health organization’s guideline for production of ABHR?

[*Probe: If “No”, ask formulation ingredients and amount to be used during production]*

Response: Yes, it is based on formulation 1 (ethanol based)

1. **Availability and supply of chemicals, materials, PPE**
2. Is there problem in the supply of ingredients, personnel protective equipment, packaging and labelling materials? *[probe: reasons of unavailability: cost or procurement difficulties]*

Response: No: there is no as such problem of ingredients, PPE and packaging and labelling materials. But, at the beginning of ABHR production, there was problem of getting the labelling materials because of sluggish purchasing process from our hospital but now it is resolved. The other issue is with respective to hydrogen peroxide as we get only 30% from the market though we need 3% which make the preparation time consuming. We have also problem of measuring jugs and beakers as there is only a liter capacity measuring jug for measuring higher volume alcohol (5 – 10 liter) and transferring to other containers.

1. What is the source of your active ingredient (ethanol or isopropyl alcohol), other chemicals and packaging and labelling materials? *[Probe: sugar industry, chemical industry, purchase from whole sale, obtained locally, imported, cost of 100 ml dispenser (for comparison)]*

Response: N**o:** The packaging and labelling materials are purchased from the local suppliers

1. Do the required equipment specifically for the production of ABHRs purchased or donated or had them already?

Response: some are purchased specifically for this purpose like chemicals, alcoholmeter and some are donated including filling containers with capacity of about 60 liters.

1. **Standard of practice**
2. Do you think that the production of ABHR solution at your facility comply the requirement or follow GCP principles? *[Probe: regular calibration and/or qualification, relevant quality control activities ABHR production (alcohol content determination, H_2_0_2_ titrimetry)]*

Response: Yes, as far as we are preparing ABHR based on the WHO guideline, we are following GCP. We have good premise which suit for compounding. We are using distilled water. There are some remaining with respect to availability of appropriate equipment like measuring jugs and absence of calibrated equipment like alcoholmeters.

1. How do you compare the ABHR solution prepared at your facility with those obtained from market? Do you have preference? Why? *(Probe: Differences in effectiveness, quality, cost (100 ml of facility produced and purchased from market)*

Response: Yes, as the healthcare providers in our hospitals prefer the inhouse product than the products obtained from the market as they explain the donated product from the market is having more gel like consistency which is not comfortable. Some other said that the product from the market is too watery showing less alcohol content than anticipated which leads failure of effectiveness. I guess that the product from the market is costlier than the in-house product.

1. If you think there is difference in any of the attributes, what are the major reasons?

*(Probe: manufacturing procedures, regulation, type of ingredients etc.)*

Response: Yes, there is also quality concern of products from market. There is expected lower quality products due to adulteration and weak control from regulatory bodies.

1. **Production capacity and future plan**
2. Do you think the manufactured ABHR solution at your facility satisfy the demand of your organization? Explain the reasons for the response?

Probe: production capacity (daily/weekly/monthly), barriers for adequate production]

Response: we are satisfying the facility’s demand by compounding 2000 bottles sanitizer of 250 ml capacity in every two weeks. Our product is delivered to more than 1000 staff in our facility and other institutions like MoH and Addis Ababa Police Department.

1. Do your organization support/sell the product to other institutions/general public?

*(Probe: agreement or contacts, the market demand)*

*Response*: Yes, we support different organizations by referring their simple official letter requesting cooperation.

1. Does your organization have a plan to continue ABHR solution production after the COVID-19 pandemic is controlled globally?

Response: Yes

1. Do you estimate/assess the demand (daily or weekly) of ABHR solution at your faculty? If yes, how? If no, why?

Response: Yes, we simply estimate the consumption by considering the amount of solution to be used once (2 ml as per WHO) and the number of times used in a day time. We are taking an average daily amount regardless of specific area of practice. For future this should be corrected as different departments may have varying burden requiring more amount. This is also evidenced by complaints from some staffs for inadequate amount of solution.

1. **Rational use of ABHR**
2. Is there proper use of dispenser bottles by the health care professionals?

*(Probe: bring them properly for refill, request new if stolen or damage of pump or cup)*

Response: No, we gave a 100 ml bottles to all for the first time. But most of them came empty handed for the second schedule and they said we lost bottles, the caps are broken and like this.

1. Do you think there is rational use of ABHR solution at the facility? Mention reasons for your response?

Response: No, there is an overall awareness problem among users. They don’t really know when and how they should use ABHR. They are using it repeatedly without touching patients or patient surroundings. There is also inappropriate amount used (more or less amount). When the number of infected individuals increased globally or nationally (when declared by media)

1. Do you consider yourself to serve as highly accessible information expert and provide evidence-based information and education about sanitizer (ABHR)?

If so, what type of information did you provide so far?

Response: No, there we haven’t involved in providing information so far through health education. But we tried to promote rational use through telegram and by indicating the direction to use in the label portion.

1. **Professional**
2. Who is involved in production in your unit?

*[Probe: pharmacists, nurses, chemists]*

Response: two pharmacists

1. Do they take training? If so, who is organize the training and when?

Response: No

1. Do you believe that pharmacy professionals are duly recognized and reimbursed for their public health services to combat COVID-19 in Ethiopia? If not, why?

Response: No, there are more things to be improved in giving attention. We didn’t take training, there is procurement bureaucracy. They don’t consider pharmacy professionals are really important (front line in combating COVID 19) like other professionals (e.g. nurses).

1. What supports are required to reimburse pharmacy professional for their service related with COVID-19 epidemic? *[Probe: accessing sanitizers, affordable price, advice in rational use, fair reimbursements]*

Response: attention/emphasis should be given to pharmacy professionals. Political decision is important in enforcing the attention to be given. The support should be from government like MOH.

1. **Best practice**
2. What best practices does your institution have in ABHR production and utilization to prevent infections (COVID-19) epidemic?

Response: We have separate compounding and packaging premise (for distribution) which minimizes traffic in the production area. We have a computerized recording system regarding distribution of ABHR products which is best way of auditing the distribution. We have also program for distribution to different units through the assigned focal persons,

1. Do you think your facility needs to be supported/strengthened/ with respect to ABHR solution production? If so, who do you think should support it? How do you think should it be supported?

*[Probe: training, material/resource]*

Response: Yes, we are compounding mainly COVID-19 related products with limited number of staff and small size measuring devices that result in repeated exposure to alcohol. The ABHR production team is not a member of COVID-19 team in the hospital that disregards pharmacists from COVID-19 related incentives. MoH should guide our hospital management to consider pharmacy professionals as part of COVID-19 team and get all related compensations and incentives for ABHR production.

**If you have further points or comments to add, we will be appreciating.**

I want to thank the management for providing specific rooms for compounding. There is no dedicated team for compounding which limits in finding solutions for the problems we are facing. Hence, it should be corrected by forming dedicated team.

**[Thank you!!!]**

**Participant #04**

| Age | 30 |
| --- | --- |
| Gender | Male |
| Highest qualification | B. Pharm |
| Current practices setting | Specialized hospitals |
| Present position | Compounding case team head |
| Year of experiences, total(year) | Five (5) years |
| Year of working experiences in the current position (years) | Two (2) years in compounding department as a head, 2 years dispenser and 3 months on ABHR compounding head. |
| When did your facility start production? | After COVID-19 Pandemic |

**Part two: key-informant guides**

1. **Nature of formulation**
2. Do you follow World Health organization’s guideline for production of ABHR?

[*Probe: If “No”, ask formulation ingredients and amount to be used during production]*

Response: Yes, in addition to the WHO guidelines we are using FMOH ABHR production guideline. And we are using Formulation one ethanol containing ABHR.

1. **Availability and supply of chemicals, materials, PPE**
2. Is there problem in the supply of ingredients, personnel protective equipment, packaging and labelling materials? *[probe: reasons of unavailability: cost or procurement difficulties]*

Response: There is no any supply shortage or problem, all items are supplied as per our demand and most PPE are supplied as a donation. packaging materials are procured from private suppliers and labelling is prepared from Ras Emeru St. Paulose printing and press wing

1. What is the source of your active ingredient (ethanol or isopropyl alcohol), other chemicals and packaging and labelling materials? *[Probe: sugar industry, chemical industry, purchase from whole sale, obtained locally, imported, cost of 100 ml dispenser (for comparison)]*

Response: The sources of packaging materials are procured from aqua and Arsho, PPE is donation, Glycerine and hydrogen peroxide from PFSA. Alcohols from Wenji and Fincha Sugar Factory

1. Do the required equipment specifically for the production of ABHRs purchased or donated or had them already?

Response: Almost all Beaker and measuring cylinder are came as donation. Plastic roto and containers are purchased

1. **Standard of practice**
2. Do you think that the production of ABHR solution at your facility comply the requirement or follow GCP principles?

*[Probe: regular calibration and/or qualification, relevant quality control activities ABHR production (alcohol content determination, H_2_0_2_ titrimetric)]*

Response: Yes, we think that we follow GCP. We request for calibration of alcoholometry but it is not done and others equipment are not calibrated. The reason is the assigned and responsible person got to upgrading education due to this their inconsistency task activities and there is lack human resource and unable to calibrate it.

1. How do you compare the ABHR solution prepared at your facility with those obtained from market? Do you have preference? Why?

*(Probe: Differences in effectiveness, quality, cost (100 ml of facility produced and purchased from market)*

Response: Concentration and cost effective or cheap

1. If you think there is difference in any of the attributes, what are the major reasons?

*(Probe: manufacturing procedures, regulation, type of ingredients etc.)*

Response: As per the standard by considering room temperature adjustment and proper procedure follow

1. Do you think the manufactured ABHR solution at your facility satisfy the demand of your organization? Explain the reasons for the response?

Probe: production capacity (daily/weekly/monthly), barriers for adequate production]

Response: Yes, we hare highly satisfied the community. 600 litter/week and we distribute for 150 caste team leader three times a week.

1. Do your organization support/sell the product to other institutions/general public?

*(Probe: agreement or contacts, the market demand)*

Response***:*** We support FMOH, policy station and commercial bank but there is no common agreement.

1. Does your organization have a plan to continue ABHR solution production after the COVID-19 pandemic is controlled globally?

Response***:*** the production of ABHR in our facility was started before COVID-19 pandemic and `will continue in the future as well since it is prepared for infection prevention program. Training on ABHR preparation was given to some staff considering continuity of production.

1. Do you estimate/assess the demand (daily or weekly) of ABHR solution at your faculty?

If yes, how? If no, why?

Response**:** Based weekly refile average estimation

1. **Rational use of ABHR**
2. Is there proper use of dispenser bottles by the health care professionals?

*(Probe: bring them properly for refill, request new if stolen or damage of pump or cup)*

Response**:** Yes, but there is quality defect dispensing bottles cap and by observing it we substitute the whole bottles. some are properly used it

1. Do you think there is rational use of ABHR solution at the facility? Mention reasons for your response?

Response**:** Pocket size user are used rationally but sometimes some persons took it for their home use. Due to the effectiveness thrust of products they took it to their home and give for others

1. Do you consider yourself to serve as highly accessible information expert and provide evidence-based information and education about sanitizer (ABHR)?

If so, what type of information did you provide so far?

Response**:** Yes, proper steps of ABHR use information, hand washing, proper use, ABHR for infection prevention rather than COVID, giving understanding on methods of preparation for pharmacist and posting of preparation steps.

1. **Professional**
2. Who is involved in production in your unit?

*[Probe: pharmacists, nurses, chemists]*

Response**:** four pharmacists

1. Do they take training? If so, who is organize the training and when?

Response**:** Yes, training is facilitated by FMOH before 4 months. But, the rest of my colleagues are not trained.

1. Do you believe that pharmacy professionals are duly recognized and reimbursed for their public health services to combat COVID-19 in Ethiopia? If not, why?

Response**:** Prevention one part of techniques for COVID-19.

1. What supports are required to reimburse pharmacy professional for their service related with COVID-19 epidemic?

*[Probe: accessing sanitizers, affordable price, advice in rational use, fair reimbursements]*

Response**:** Due to the toxicity of the ingredients per dime or financial incentives. training, hiring human resources, per dime/ incentives.

1. **Best practice**
2. What best practices does your institution have in ABHR production and utilization to prevent infections (COVID-19) epidemic?

Response**:** Good practices, assigning only one person on ABHR production can facilitates the production and free the case team leaders from other burdens. Thus, this could be good practices. Starting of ABHR immediately without any incentives and agreement would be best practices.

1. Do you think your facility needs to be supported/strengthened/ with respect to ABHR solution production? If so, who do you think should support it? How do you think should it be supported?

*[Probe: training, material/resource]*

Response**:** I believe MoH should provide in-service training on ABHR production and provide consistent follow-up to maintain the sustainability of ABHR production and compounding services. Hospital administrators should pay attention for the compounding service by allocating premises and recruiting sufficient personnel.

Comments: Giving Training on Dermatology compounding and following the activities through report mechanism and supporting the institutions

**[Thank you!!!]**

**Participant #05**

| Age | 32 |
| --- | --- |
| Gender | Female |
| Highest qualification | B. Pharm |
| Current practices setting | Specialized hospitals |
| Present position | Compounding coordinator |
| Year of experiences, total(year) | 7 years |
| Year of working experiences in the current position (years) | 5 months |
| When did your facility start production? | After COVID-19 Pandemic |

1. **Nature of formulation**
2. Do you follow World Health organization’s guideline for production of ABHR? [*Probe: If “No”, ask formulation ingredients and amount to be used during production]*

Response: Who guideline formula 1 and the ingredients are Alcohol 99 %, 3 % hydrogen peroxide and glycerine.

1. **Availability and supply of chemicals, materials, PPE**
2. Is there problem in the supply of ingredients, personnel protective equipment, packaging and labelling materials? *[probe: reasons of unavailability: cost or procurement difficulties]*

Response: During starting of the production of ABHR for our staff, we have got scarcity of Alcohol and Problem of Bottles and self-adhesive labelling printing machine but now we are using paper in printed way. Reason: *starting* of the production as an emergency and limited supplier (i.e. there is only 2 suppliers. Example National alcohol factory)

1. What is the source of your active ingredient (ethanol or isopropyl alcohol), other chemicals and packaging and labelling materials? *[Probe: sugar industry, chemical industry, purchase from whole sale, obtained locally, imported, cost of 100 ml dispenser (for comparison)]*

Response: Private Suppliers. 100 ml bottles 2-5 birr and sugar corporation for ethanol

1. Do the required equipment specifically for the production of ABHRs purchased or donated or had them already?

Response: We purchased large Mixer and large plastic tanker (Rotto). we expand production house for production as per the standard but we never get any kind of equipment as donation except sugar corporation ethanol

1. **Standard of practice**
2. Do you think that the production of ABHR solution at your facility comply the requirement or follow GCP principles? *[Probe: regular calibration and/or qualification, relevant quality control activities ABHR production (alcohol content determination, H_2_0_2_ titrimetry)]*

Response: Based criteria of WHO we checked the quality of starting alcohols by alcoholmeters and keep sanitization of products in safe places, check the final products

Before 5 month we calibrated only alcoholometer.

1. How do you compare the ABHR solution prepared at your facility with those obtained from market? Do you have preference? Why? *(Probe: Differences in effectiveness, quality, cost (100 ml of facility produced and purchased from market)*

Response: In terms of quality, we checked marketed products with our products by using alcoholmeters and in terms of cost our product is fair i.e. 50 ml 60 birr.

1. If you think there is difference in any of the attributes, what are the major reasons?

*(Probe: manufacturing procedures, regulation, type of ingredients etc.)*

Response: Quality of ingredients.

1. **Production capacity and future plan**
2. Do you think the manufactured ABHR solution at your facility satisfy the demand of your organization? Explain the reasons for the response? Probe: production capacity (daily/weekly/monthly), barriers for adequate production]

Response: Yes, after COVID -19 by counting the staff number we estimate the demand of ABHR.

1. Do your organization support/sell the product to other institutions/general public? *(Probe: agreement or contacts, the market demand)*

Response: We Just deliver ABHR for community as a promotion mechanism.

1. Does your organization have a plan to continue ABHR solution production after the COVID-19 pandemic is controlled globally?

Response: Yes, we continue because it is considered as self-hygiene practices

1. Do you estimate/assess the demand (daily or weekly) of ABHR solution at your faculty? If yes, how? If no, why?

Response: Yes, by simply counting the staff and adjust the weekly /daily demand.

1. **Rational use of ABHR**
2. Is there proper use of dispenser bottles by the health care professionals?

*(Probe: bring them properly for refill, request new if stolen or damage of pump or cup)*

Response: There is no proper use of dispensing bottles we just deliver new bottles. We are bulk self-refilling methods. Reason: due you to the COVID-19 situation and cross-contamination risk we never re-use the bottles and due to human resources and disinfectant we cannot recycle the bottle through different disinfection methods.

1. Do you think there is rational use of ABHR solution at the facility? Mention reasons for your response?

Response: no response

1. Do you consider yourself to serve as highly accessible information expert and provide evidence-based information and education about sanitizer (ABHR)? If so, what type of information did you provide so far?

Response: Yes, after COVID we deliver on proper use of ABHR as an individual level.

1. **Professional**
2. Who is involved in production in your unit? *[Probe: pharmacists, nurses, chemists]*

Response: pharmacists

1. Do they take training? If so, who is organize the training and when?

Response: Only one person got training

1. Do you believe that pharmacy professionals are duly recognized and reimbursed for their public health services to combat COVID-19 in Ethiopia? If not, why?

Response: Yes, without any additional facilities we are working but we are not recognized.

We get reimbursement inform of duty payment only.

1. What supports are required to reimburse pharmacy professional for their service related with COVID-19 epidemic? *[Probe: accessing sanitizers, affordable price, advice in rational use, fair reimbursements]*

Response: Paying for risk due to the toxicity of alcohols should be considered and delivering the ABHR with Affordable price

1. **Best practice**
2. What best practices does your institution have in ABHR production and utilization to prevent infections (COVID-19) epidemic?

Response: We are working through standard strictly and using pharmacy staff inform of rotation to gain knowledge and work effectively,

1. Do you think your facility needs to be supported/strengthened/ with respect to ABHR solution production? If so, who do you think should support it? How do you think should it be supported?

*[Probe: training, material/resource]*

Response: Mixer support from FMOH, technology update, continues follow up and interlinked with institutions with the aid of FMOH.

**If you have further points or comments to add, we will be appreciating.**

The institutions should expand the service in producing of detergent and others quality products.

**[Thank you!]**

**Participant #06**

| Age | 35 |
| --- | --- |
| Gender | Male |
| Highest qualification | B. Pharm |
| Current practices setting | General hospitals |
| Present position | Compounding case team head |
| Year of experiences, total(year) | Five (5) years |
| Year of working experiences in the current position (years) | 4 months |
| When did your facility start production? | After COVID-19 Pandemic |

1. **Nature of formulation**
2. Do you follow World Health organization’s guideline for production of ABHR?

[*Probe: If “No”, ask formulation ingredients and amount to be used during production]*

Response: Yes, the procedures and type of ingredients for ABHR production are as per WHO guidelines. However, WHO puts QC/QA parameters and we are not fully following such parameters.

1. **Availability and supply of chemicals, materials, PPE**
2. Is there problem in the supply of ingredients, personnel protective equipment, packaging and labelling materials? *[probe: reasons of unavailability: cost or procurement difficulties]*

Response: There is no any supply shortage or problem, all items are supplied as per our demand and most PPE are supplied as a donation. packaging materials are procured from private suppliers and labelling is prepared from Ras Emeru St. Paulose printing and press wing

1. What is the source of your active ingredient (ethanol or isopropyl alcohol), other chemicals and packaging and labelling materials?

*[Probe: sugar industry, chemical industry, purchase from whole sale, obtained locally, imported, cost of 100 ml dispenser (for comparison)]*

Response: Ethanol from AARHB, Glycerol and hydrogen peroxide from PFSA, packaging materials purchased from wholesales and labelling are self-designed printing.

1. Do the required equipment specifically for the production of ABHRs purchased or donated or had them already?

Response: There is no any purchased equipment for the production of ABHR and we used already available equipment in store, which is purchased before

1. **Standard of practice**
2. Do you think that the production of ABHR solution at your facility comply the requirement or follow GCP principles?

*[Probe: regular calibration and/or qualification, relevant quality control activities ABHR production (alcohol content determination, H_2_0_2_ titrimetric)]*

Response: No because the room is assigned only for production of ABHR. Thus, the room is not convenient for QC/QA, storage of ABHR and all ABHR production activities are performed at one place. Due to this we can say we are not following GCP

1. How do you compare the ABHR solution prepared at your facility with those obtained from market? Do you have preference? Why? *(Probe: Differences in effectiveness, quality, cost (100 ml of facility produced and purchased from market)*

Response: The hospital stops ABHR production for 2 months because of the donation of sanitizer from different institutions. However, different comments were raised from the staffs with regard to odour and the prepared type of ABHR has good Oder than the donated types

1. If you think there is difference in any of the attributes, what are the major reasons? *(Probe: manufacturing procedures, regulation, type of ingredients etc.)*

Response: The major reason of differences is we are using ingredients for ABHR production is as per the standard of WHO

1. Do you think the manufactured ABHR solution at your facility satisfy the demand of your organization? Explain the reasons for the response? Probe: production capacity (daily/weekly/monthly), barriers for adequate production]

Response: We couldn’t satisfy the demand of the staff. That is why additional sanitizers were requested and collected from donors. Main reasons of failure to compound ABHR at our facility regularly are inconsistent supply of ingredients and lack of adequate man power*.*

1. Do your organization support/sell the product to other institutions/general public?

*(Probe: agreement or contacts, the market demand)*

Response***:*** we have no any formal agreement or contact to support or sell

1. Does your organization have a plan to continue ABHR solution production after the COVID-19 pandemic is controlled globally?

Response***:*** Yes, if the compounding room is in good setup and fulfilling necessary equipment, we can produce it.

1. Do you estimate/assess the demand (daily or weekly) of ABHR solution at your faculty?

If yes, how? If no, why?

Response**:** There are no exceptional demand assessment methods but based on the IFRR report of the case team head we simply estimate the demand of ABHR.

1. **Rational use of ABHR**
2. Is there proper use of dispenser bottles by the health care professionals?

*(Probe: bring them properly for refill, request new if stolen or damage of pump or cup)*

Response**:** There is no rational use of ABHR packing in both donated type and gifted by pharmacy. Due to the inconvenience to handle and spray, they shifted/changed it to other convenient type packing cup.

1. Do you think there is rational use of ABHR solution at the facility? Mention reasons for your response?

Response**:** There is no rational use of ABHR. It is distributed by head of department and this distribution manner is inappropriate. There is inappropriate use of ABHR at the right time and in right places. This is due to lack of awareness.

1. Do you consider yourself to serve as highly accessible information expert and provide evidence-based information and education about sanitizer (ABHR)?

If so, what type of information did you provide so far?

Response**:** Yes, we gave information on rational use of ABHR and hand washing methods on right time and right places through banner.

1. **Professional**
2. Who is involved in production in your unit? *[Probe: pharmacists, nurses, chemists]*

Response**:** pharmacists

1. Do they take training? If so, who is organize the training and when?

Response**:** No.

1. Do you believe that pharmacy professionals are duly recognized and reimbursed for their public health services to combat COVID-19 in Ethiopia? If not, why?

Response**:** FMOH, AARHB and the pharmacy department are not promoting the pharmacy activities with regard to COVID-19 prevention through ABHR production. Thus, due to this the community are not giving recognition and are not seen as frontline activities. EPA and FMOH collaborate advocates pharmacy intervention on COVID-19.

1. What supports are required to reimburse pharmacy professional for their service related with COVID-19 epidemic?

*[Probe: accessing sanitizers, affordable price, advice in rational use, fair reimbursements]*

Response**:** Recognition from FMOH, incentives and capacity building based on the performances of the institutions.

1. **Best practice**
2. What best practices does your institution have in ABHR production and utilization to prevent infections (COVID-19) epidemic?

Response**:** Giving lecture on ABHR Vs Hand hygiene for community is our best practices.

1. Do you think your facility needs to be supported/strengthened/ with respect to ABHR solution production?

If so, who do you think should support it? How do you think should it be supported?

*[Probe: training, material/resource]*

Response**:** Training, delivering UpToDate information materials, financial and material support from FMOH

Comments: Before proceeding the ABHR preparation it was better to give training on ABHR production for the directors, CEO and case team heads.

**[Thank you!!!]**

**Participant: #07**

| Age (years) | 27 |
| --- | --- |
| Gender | Female |
| Highest Qualification | BPharm |
| Current practice setting | Specialized Hospital |
| Present position | Compounding coordinator |
| Year of experience, total (years) | 5 |
| Years of working experience in the current position (years) | 6 months |
| When did your facility start production? | After COVID-19 Pandemic |

1. **Nature of formulation**
2. Do you follow World Health organization’s guideline for production of ABHR? [*Probe: If “No”, ask formulation ingredients and amount to be used during production]*

Response: Yes, it is based on formulation 1 (ethanol based)

1. **Availability and supply of chemicals, materials, PPE**
2. Is there problem in the supply of ingredients, personnel protective equipment, packaging and labelling materials? *[probe: reasons of unavailability: cost or procurement difficulties]*

Response: No: there is no as such problem of ingredients, PPE and packaging and labelling materials. To start we obtained chemicals (ethanol, H2O2, Glycerol) from wholesalers. Now, we are getting ethanol from sugar factory. Glycerol and H2O2 are easily obtained from EPSA.

We have sufficient number of the required PPE (like facemask, goggle etc). we don’t have should cover and head cover.

Packaging and labelling materials: we are currently using self-printed labelling materials having the required information. We are planning to purchase self-adhesive labelling materials from market in near future. Regarding to packaging bottles, we are now using pocket size (125 ml to be self-refilled) and 1 liter capacity bottles. We couldn’t find other capacity bottles from market (e.g. 250 and 500 ml) and waiting from tender.

1. What is the source of your active ingredient (ethanol or isopropyl alcohol), other chemicals and packaging and labelling materials? *[Probe: sugar industry, chemical industry, purchase from whole sale, obtained locally, imported, cost of 100 ml dispenser (for comparison)]*

Response: initially we purchased the ethanol from wholesale by tenders. Later on we received alcohol (4000 liter) from Metehara Sugar Factory by means of MOH. H2O2 and glycerol from EPSA. The packaging bottles were purchased from the local wholesalers.

1. Do the required equipment specifically for the production of ABHRs purchased or donated or had them already?

Response: We have most required materials (e.g. measuring cylinders, funnels and others) as there is compounding of dermatological service already. some are purchased specifically for this purpose like storing tankers, alcoholmeter and wall thermometers

1. **Standard of practice**
2. Do you think that the production of ABHR solution at your facility comply the requirement or follow GCP principles? *[Probe: regular calibration and/or qualification, relevant quality control activities ABHR production (alcohol content determination, H_2_0_2_ titrimetry)]*

Response: Yes, in a certain degree, it is in a good condition. Yet there are some things to be improved like storage of chemicals, packaging materials and packed products, limited space for packaging and labelling operation. We have separate team for QC activity but not fully functional.

1. How do you compare the ABHR solution prepared at your facility with those obtained from market? Do you have preference? Why? *(Probe: Differences in effectiveness, quality, cost (100 ml of facility produced and purchased from market)*

Response: Yes, as the healthcare providers in our hospitals prefer the inhouse product than the products obtained from the market as they explain the donated product from the market is having more gel like consistency which is not comfortable. Some other said that the product from the market is too watery showing less alcohol content than anticipated which leads failure of effectiveness. I guess that the product from the market is costlier than the in-house product.

1. If you think there is difference in any of the attributes, what are the major reasons?

*(Probe: manufacturing procedures, regulation, type of ingredients etc.)*

Response: Yes, there is also quality concern of products from market. There is expected lower quality products due to adulteration and weak control from regulatory bodies.

1. **Production capacity and future plan**
2. Do you think the manufactured ABHR solution at your facility satisfy the demand of your organization? Explain the reasons for the response? Probe: production capacity (daily/weekly/monthly), barriers for adequate production]

Response: Yes, we are satisfying the facility demand. Furthermore, we are supporting other institutions like MOH, AA Police department and other health care facilities in the city. We have scheduled the production time to be once in every two weeks (2000 bottles of 250 ml for each staff). More than 1000 staffs are there in the facility.

1. Do your organization support/sell the product to other institutions/general public?

*(Probe: agreement or contacts, the market demand)*

Response: Yes, we support different organizations by referring their simple official letter requesting cooperation.

1. Does your organization have a plan to continue ABHR solution production after the COVID-19 pandemic is controlled globally?

Response: Yes

1. Do you estimate/assess the demand (daily or weekly) of ABHR solution at your faculty? If yes, how? If no, why?

Response: Yes, we simply estimate the consumption by considering the amount of solution to be used once (2 ml as per WHO) and the number of times used in a day time. We are taking an average daily amount regardless of specific area of practice. For future this should be corrected as different departments may have varying burden requiring more amount. This is also evidenced by complaints from some staffs for inadequate amount of solution.

1. **Rational use of ABHR**
2. Is there proper use of dispenser bottles by the health care professionals?

*(Probe: bring them properly for refill, request new if stolen or damage of pump or cup)*

Response: No, we gave a 100 ml bottles to all for the first time. But most of them came empty handed for the second schedule and they said we lost bottles, the caps are broken and like this.

1. Do you think there is rational use of ABHR solution at the facility? Mention reasons for your response?

Response: No, there is an overall awareness problem among users. They don’t really know when and how they should use ABHR. They are using it repeatedly without touching patients or patient surroundings. There is also inappropriate amount used (more or less amount). When the number of infected individuals increased globally or nationally (when declared by media)

1. Do you consider yourself to serve as highly accessible information expert and provide evidence-based information and education about sanitizer (ABHR)? If so, what type of information did you provide so far?

Response: No, there we haven’t involved in providing information so far through health education. But we tried to promote rational use through telegram and by indicating the direction to use in the label portion.

1. **Professional**
2. Who is involved in production in your unit? *[Probe: pharmacists, nurses, chemists]*

Response: two pharmacists

1. Do they take training? If so, who is organize the training and when?

Response: No

1. Do you believe that pharmacy professionals are duly recognized and reimbursed for their public health services to combat COVID-19 in Ethiopia? If not, why?

Response: No, there are more things to be improved in giving attention. We didn’t take training, there is procurement bureaucracy.

1. What supports are required to reimburse pharmacy professional for their service related with COVID-19 epidemic? *[Probe: accessing sanitizers, affordable price, advice in rational use, fair reimbursements]*

Response: attention/emphasis should be given to pharmacy professionals. Political decision is important in enforcing the attention to be given. The support should be from government like MOH.

1. **Best practice**
2. What best practices does your institution have in ABHR production and utilization to prevent infections (COVID-19) epidemic?

Response: We have separate compounding and packaging premise (for distribution) which minimizes traffic in the production area. We have a computerized recording system regarding distribution of ABHR products which is best way of auditing the distribution. We have also program for distribution to different units through the assigned focal persons,

1. Do you think your facility needs to be supported/strengthened/ with respect to ABHR solution production? If so, who do you think should support it? How do you think should it be supported?

*[Probe: training, material/resource]*

Response: Yes, we are compounding mainly COVID related products (ABHR and 70% alcohol) with only two assigned persons which are not incorporated in COVID team. We are repeatedly exposed for the alcohol because of less number involved and tedious compounding process mainly from lack of large measuring devices. It should be corrected. MOH personnel should convince our management persons to consider pharmacy professional engaged in compounding ABHR as COVID team and get all the advantages like others.

**If you have further points or comments to add, we will be appreciating.**

I want to thank the management for providing specific rooms for compounding. There is no dedicated team for compounding which limits in finding solutions for the problems we are facing. Hence, it should be corrected by forming dedicated team.

**[Thank you!!!]**

**Participant #08**

| Age (years) | 27 |
| --- | --- |
| Gender | Male |
| Highest Qualification | BPharm |
| Current practice setting | Specialized Hospital |
| Present position | Compounding coordinator |
| Year of experience, total (years) | 5 |
| Years of working experience in the current position (years) | 3 months |
| When did your facility start production? | After COVID-19 Pandemic |

1. **Nature of formulation**
2. Do you follow World Health organization’s guideline for production of ABHR?

[*Probe: If “No”, ask formulation ingredients and amount to be used during production]*

Response: Yes, it is based on formulation 1 (ethanol based)

1. **Availability and supply of chemicals, materials, PPE**
2. Is there problem in the supply of ingredients, personnel protective equipment, packaging and labelling materials? *[probe: reasons of unavailability: cost or procurement difficulties]*

Response: Regarding to ethanol, there was problem of getting it for initiating the production of ABHR. But once we started, we didn’t face supply shortage. We have received two times for production and it’s sufficient for our demand.

We couldn’t get the packaging bottles and labelling materials so far because of bureaucratic process for purchasing

PPE: No problem of having it

1. What is the source of your active ingredient (ethanol or isopropyl alcohol), other chemicals and packaging and labelling materials?

*[Probe: sugar industry, chemical industry, purchase from whole sale, obtained locally, imported, cost of 100 ml dispenser (for comparison)]*

Response: For ethanol, it’s from Wonji sugar factory, for hydrogen peroxide and glycerol, it is from EPSA and packaging and labelling material: from the local plastic factories

1. Do the required equipment specifically for the production of ABHRs purchased or donated or had them already?

Response**:** some are purchased specifically for this purpose like chemicals, mixing containers and some are donated including measuring cylinder, funnels etc.

1. **Standard of practice**
2. Do you think that the production of ABHR solution at your facility comply the requirement or follow GCP principles?

*[Probe: regular calibration and/or qualification, relevant quality control activities ABHR production (alcohol content determination, H_2_0_2_ titrimetry)]*

Response: Yes, though there are some limitations in requirements, we are following GCP. Because, we have separate room, proper attire system, we have some required production equipment. We don’t have QC operations.

**,** as far as we are preparing ABHR based on the WHO guideline, we are following GCP. We have good premise which suit for compounding. We are using distilled water. There are some remaining with respect to availability of appropriate equipment like measuring jugs and absence of calibrated equipment like alcoholmeters.

1. How do you compare the ABHR solution prepared at your facility with those obtained from market? Do you have preference? Why?

*(Probe: Differences in effectiveness, quality, cost (100 ml of facility produced and purchased from market)*

Response: No difference in most domestic products from market**,** as far as they are preparing ABHR based on the WHO guideline, there is no difference between the products in market and inhouse product. But there are few products with thick consistency because of gelling agent. Generally, the cost of products from market is a bit higher than our product.

1. If you think there is difference in any of the attributes, what are the major reasons?

*(Probe: manufacturing procedures, regulation, type of ingredients etc.)*

Response: No.

1. **Production capacity and future plan**
2. Do you think the manufactured ABHR solution at your facility satisfy the demand of your organization? Explain the reasons for the response?

Probe: production capacity (daily/weekly/monthly), barriers for adequate production]

*Response*: Yes, we are satisfying the facility demand as far as there is no interruption of ethanol supply. We are producing two times in a week with a total of about 600 liter per week. There are about 500 workers in our hospital.

1. Do your organization support/sell the product to other institutions/general public?

*(Probe: agreement or contacts, the market demand)*

Response: Yes, we support some institutions once by referring their simple official letter requesting cooperation.

1. Does your organization have a plan to continue ABHR solution production after the COVID-19 pandemic is controlled globally?

*Response*: Yes, I think so if they fulfil the room requested for it.

1. Do you estimate/assess the demand (daily or weekly) of ABHR solution at your faculty?

If yes, how? If no, why?

*Response*: Yes, we simply estimate the consumption by considering complains from initial delivery. Now, we are providing with the estimated consumption of 500 ml per person per week.

1. **Rational use of ABHR**
2. Is there proper use of dispenser bottles by the health care professionals?

*(Probe: bring them properly for refill, request new if stolen or damage of pump or cup)*

*Response*: Yes, I think so**,** we gave a 125 ml bottles to all for the first time. After that they are using these bottles and their own personal bottles for refilling (by themselves) by taking ABHR (using department heads) and there is no complain about bottles so far.

1. Do you think there is rational use of ABHR solution at the facility? Mention reasons for your response?

Response: Yes, there is rational use of sanitizers by health workers in our facility because they have awareness on when and how much to use. We have also reached the users through focal person of each unit during product distribution.

1. Do you consider yourself to serve as highly accessible information expert and provide evidence-based information and education about sanitizer (ABHR)? If so, what type of information did you provide so far?

Response: I don’t think that pharmacists are the only information providers on sanitizers’ rational use. This time everybody knows about sanitizers and has sufficient information on its rational use as this product is repeatedly advertised to the public through different media.

1. **Professional**
2. Who is involved in production in your unit? *[Probe: pharmacists, nurses, chemists]*

Response: three pharmacists

1. Do they take training? If so, who is organize the training and when?

Response: Yes, by MOH in December, 2019.

1. Do you believe that pharmacy professionals are duly recognized and reimbursed for their public health services to combat COVID-19 in Ethiopia? If not, why?

Response: I think so, the importance of sanitizer by the government is well known as pharmacists are producing it. And attention is given for pharmacist but it is not with the expected one.

1. What supports are required to reimburse pharmacy professional for their service related with COVID-19 epidemic? *[Probe: accessing sanitizers, affordable price, advice in rational use, fair reimbursements]*

Response: more attention/emphasis should be given to pharmacy professionals as they are also front-line workers with the current pandemic disease. Protection of pharmacists using appropriate PPE should be considered. Payments for risk should be settled for them as well.

1. **Best practice**
2. What best practices does your institution have in ABHR production and utilization to prevent infections (COVID-19) epidemic?

Response: We are producing in large capacity (>300 liter) and participate other personnel for ABHR distribution to different wards.

1. Do you think your facility needs to be supported/strengthened/ with respect to ABHR solution production? If so, who do you think should support it? How do you think should it be supported? *[Probe: training, material/resource]*

Response: Yes, but mainly we need support from our management (self-support) personnel by providing appropriate premise for compounding and storage and fulfilling all the required materials for production (e.g. alcoholmeter, measuring devices etc)

**If you have further points or comments to add, we will be appreciating. no**

**[Thank you!!!]**

**Participant # 09**

| Age (years) | 34 |
| --- | --- |
| Gender | Male |
| Highest Qualification | BPharm |
| Current practice setting | Specialized Hospital |
| Present position | Compounding coordinator |
| Year of experience, total (years) | 7 years |
| Years of working experience in the current position (years) | 2 years |
| When did your facility start production? | Before COVID-19 Pandemic |

1. **Nature of formulation**
2. Do you follow World Health organization’s guideline for production of ABHR?

[*Probe: If “No”, ask formulation ingredients and amount to be used during production]*

Response: Yes, it is based on formulation 1 (ethanol based) since recent time but previously it was prepared by simple mixing of ethanol (70%) and glycerol

1. **Availability and supply of chemicals, materials, PPE**
2. Is there problem in the supply of ingredients, personnel protective equipment, packaging and labelling materials? *[probe: reasons of unavailability: cost or procurement difficulties]*

Response: Yes, there are problems**:**

**PPE**: there are no full supply of the required PPE e.g. head and shoe covers but others gown, eye goggles and gloves are available. Ingredients: ethanol are obtained from sugar industries through MOH, Glycerine and H2O2 from EPSA, Packaging bottles: the supply is not satisfactory as the supply from plastic factories is limited. Generally, these supplies are obtained from purchasing or donation

1. What is the source of your active ingredient (ethanol or isopropyl alcohol), other chemicals and packaging and labelling materials? *[Probe: sugar industry, chemical industry, purchase from whole sale, obtained locally, imported, cost of 100 ml dispenser (for comparison)]*

Response: Ingredients: ethanol is obtained from sugar industries through MOH, Glycerine and H2O2 from EPSA, Packaging bottles: the supply is not satisfactory as the supply from plastic factories is limited.

1. Do the required equipment specifically for the production of ABHRs purchased or donated or had them already?

Response: all were available at the hospital except alcoholmeter and standardized pocket size packaging bottles purchased for production of WHO recommended ABHR.

1. **Standard of practice**
2. Do you think that the production of ABHR solution at your facility comply the requirement or follow GCP principles? *[Probe: regular calibration and/or qualification, relevant quality control activities ABHR production (alcohol content determination, H_2_0_2_ titrimetry)]*

Response: I think the compounding practice in our site lack some of the basic requirements for GCP. For example, there is no well-established premise and we are not assuring the strength of hydrogen peroxide and glycerol used for the production of ABHR solution. Equipment like alcoholmeter and thermometer are not regularly calibrated as well.

1. How do you compare the ABHR solution prepared at your facility with those obtained from market? Do you have preference? Why? *(Probe: Differences in effectiveness, quality, cost (100 ml of facility produced and purchased from market)*

Response: Yes, there is difference, the facility-based products have better quality than those available in the market (as it contains other additives (e.g. coloring agent) that may affect its effectiveness). The feedback from customers further indicates our product’s preference (as the required strength of ethanol is available) preference than others (because of unique flavor and color). Our product is cheaper than the commercial products (100ml is for sale by 30 birr Vs 60 birr for the commercial product)

1. If you think there is difference in any of the attributes, what are the major reasons? *(Probe: manufacturing procedures, regulation, type of ingredients etc.)*

Response: Yes, this is due to poor quality of these products which is resulted from the nature and quantity of ingredients.

1. **Production capacity and future plan**
2. Do you think the manufactured ABHR solution at your facility satisfy the demand of your organization? Explain the reasons for the response? *Probe: production capacity (daily/weekly/monthly), barriers for adequate production]*

Response: NO, we don’t satisfy the facility demand as there are some complaints from the facility. The reasons include lack of sufficient staff assigned for the production. There is also limitation on teaching rational use of products (awareness creation problem).

1. Do your organization support/sell the product to other institutions/general public?

(Probe: agreement or contacts, the market demand)

Response: Yes, we support different organizations by referring their simple official letter requesting cooperation (E.g. from CBE)

1. Does your organization have a plan to continue ABHR solution production after the COVID-19 pandemic is controlled globally?

Response: Definitely, as we have started production before COVID 19 and it is part of hospital’s IPC program, it will continue even though this pandemic will be under control.

1. Do you estimate/assess the demand (daily or weekly) of ABHR solution at your faculty?

If yes, how? If no, why?

Response: Not that much, it is based on rough estimation of ABHR consumption with the number of staff. We are accepting the refill period from environmental personnel

1. **Rational use of ABHR**
2. Is there proper use of dispenser bottles by the health care professionals?

*(Probe: bring them properly for refill, request new if stolen or damage of pump or cup)*

Response: No. Most of them came empty handed for the second schedule and they said we lost bottles; the caps are broken or replaced which do not fit the bottles and like this.

1. Do you think there is rational use of ABHR solution at the facility? Mention reasons for your response?

Response: No, some of the hospital staff don’t know when and how they should use ABHR solution. E.g., they are using it to clean their hands after taking meals, for disinfecting masks and bags, using on wet hands immediately after hand-washing*.*

1. Do you consider yourself to serve as highly accessible information expert and provide evidence-based information and education about sanitizer (ABHR)? If so, what type of information did you provide so far?

Response: Yes, there we provide as much information as we can through DIC (telegram, posting on notice board, leaflets). But as we are in technology era, everybody has chance of

1. Who is involved in production in your unit? *[Probe: pharmacists, nurses, chemists]*

Response: All are pharmacy professionals

1. Do they take training? If so, who is organize the training and when?

Response**:** Only the compounding coordinator (that is me) took training. The training was organized by ministry of Health.

1. Do you believe that pharmacy professionals are duly recognized and reimbursed for their public health services to combat COVID-19 in Ethiopia? If not, why?

Response**:** Even though the pharmacy professionals are actively involved in COVID-19 prevention activities, I believe we are disregarded by the management for the appropriate compensations. The pharmacists involved in compounding of ABHR are not compensated for their duties. On the other way, COVID-19 made the profession to be demanded since ABHR production is highly required.

1. What supports are required to reimburse pharmacy professional for their service related with COVID-19 epidemic? *[Probe: accessing sanitizers, affordable price, advice in rational use, fair reimbursements]*

Response**:** compensation for risk of ABHR (since alcohol and H2o2 are both toxic) like other healthcare professionals.

1. **Best practice**
2. What best practices does your institution have in ABHR production and utilization to prevent infections (COVID-19) epidemic?

Response**:** Distribution of ABHR for free (3000 so far) for disabled persons and schools.

1. Do you think your facility needs to be supported/strengthened/ with respect to ABHR solution production? If so, who do you think should support it? How do you think should it be supported?

*[Probe: training, material/resource]*

Response**:** WHO formula is not the only formula and it would be better if institutions can bring a better formula with evidence.

**[Thank you!!!]**

**Participant #10**

| Age | 33 |
| --- | --- |
| Gender | Male |
| Highest qualification | B. Pharm |
| Current practices setting | General hospitals |
| Present position | Compounding case team head |
| Year of experiences, total(year) | Nine (9) years |
| Year of working experiences in the current position (years) | 2 months |
| When did your facility start production? | After COVID-19 Pandemic |

1. **Nature of formulation**
2. Do you follow World Health organization’s guideline for production of ABHR?

[*Probe: If “No”, ask formulation ingredients and amount to be used during production]*

Response: Yes, the procedures and type of ingredients for ABHR production are as per WHO guidelines. However, we are not fulfilling the WHO criteria. For example, we are using Tape water for production of ABHR. Thus, we are not strictly following the WHO ABHR production principles.

1. **Availability and supply of chemicals, materials, PPE**
2. Is there problem in the supply of ingredients, personnel protective equipment, packaging and labelling materials? *[probe: reasons of unavailability: cost or procurement difficulties]*

Response: Yes, there is scarcity of PPE specially hair cover and shoe cover. There is also a problem on supply of dispensing bottles, labelling. The main reasons are unable to give attention for the production of Compounding services and lack of commitment among professionals.

1. What is the source of your active ingredient (ethanol or isopropyl alcohol), other chemicals and packaging and labelling materials?

*[Probe: sugar industry, chemical industry, purchase from whole sale, obtained locally, imported, cost of 100 ml dispenser (for comparison)]*

Response: The main supply of raw materials like Glycerol and hydrogen peroxide is from PFSA but ethanol is obtained from AARHB. The packaging materials specially 100 ml are donated from other donors for other purpose, but we are shifted to packing of ABHR. Labelling materials are simply printing format

1. Do the required equipment specifically for the production of ABHRs purchased or donated or had them already?

Response: The equipment is already available in store but some are donated for other programs like plastic jerrican 20 ml

1. **Standard of practice**
2. Do you think that the production of ABHR solution at your facility comply the requirement or follow GCP principles? *[Probe: regular calibration and/or qualification, relevant quality control activities ABHR production (alcohol content determination, H_2_0_2_ titrimetry)]*

Response: No there is no an equipment for product quality control check like Alcoholometer, hydrogen peroxide strips. Even the is no sufficient man power for QC/QA and production

1. How do you compare the ABHR solution prepared at your facility with those obtained from market? Do you have preference? Why? *(Probe: Differences in effectiveness, quality, cost (100 ml of facility produced and purchased from market)*

Response: We never compare our ABHR products with other in terms of cost, quality. because we are not widely produced

1. If you think there is difference in any of the attributes, what are the major reasons?

*(Probe: manufacturing procedures, regulation, type of ingredients etc.)*

Response: Due to limited ABHR production we never compare our products

1. Do you think the manufactured ABHR solution at your facility satisfy the demand of your organization? Explain the reasons for the response? *Probe: production capacity (daily/weekly/monthly), barriers for adequate production]*

Response: No, we are not satisfying the demand of our organization because the unavailability of raw materials like FBC water, packing bottles and labelling materials, there is production interruption and we produce 90 litter per 2 weeks.

1. Do your organization support/sell the product to other institutions/general public? *(Probe: agreement or contacts, the market demand)*

Response***:*** We delivered freely for AARHB, MOH as a support. But there is no formal support or agreement with our institutions

1. Does your organization have a plan to continue ABHR solution production after the COVID-19 pandemic is controlled globally?

Response***:*** Yes, if training, incentives are given and sufficient equipment and raw materials are provided we can produce in continuous manner

1. Do you estimate/assess the demand (daily or weekly) of ABHR solution at your faculty?

If yes, how? If no, why?

Response**:** Yes, our methods of demand estimation for ABHR is based on the demand of Caste team head demands and we distribute the products for case team head. This a method to estimate demands that we have.

1. **Rational use of ABHR**
2. Is there proper use of dispenser bottles by the health care professionals?

*(Probe: bring them properly for refill, request new if stolen or damage of pump or cup)*

Response**:** since we have no sufficient packaging bottles for our community, we cannot substitute the damaged bottles. This is communicated to users especially for front-line staffs. Therefore, they are handling packaging bottles properly.

1. Do you think there is rational use of ABHR solution at the facility? Mention reasons for your response?

Response**:** No, because ABHR is quantified and distributed by case team head. This type of distribution manners can easily wastage the products and cannot easily trace accountability.

1. Do you consider yourself to serve as highly accessible information expert and provide evidence-based information and education about sanitizer (ABHR)? If so, what type of information did you provide so far?

Response**:** Yes, we gave rational use of ABHR and HH for hospital community by DIS and post different poster inside and outside the compound.

1. **Professional**
2. Who is involved in production in your unit? *[Probe: pharmacists, nurses, chemists]*

Response: only pharmacists

1. Do they take training? If so, who is organize the training and when?

Response**:** No.

1. Do you believe that pharmacy professionals are duly recognized and reimbursed for their public health services to combat COVID-19 in Ethiopia? If not, why?

Response**:** No. There is no any incentive /reimbursement for pharmacy professionals specially on ABHR production personnel relatively with other professionals those who are working on COVID-19. The main reason is nationally unable to announce the activities of pharmacy service during the prevention of COVID-19 pandemic.

1. What supports are required to reimburse pharmacy professional for their service related with COVID-19 epidemic? *[Probe: accessing sanitizers, affordable price, advice in rational use, fair reimbursements]*

Response**:** Maintain Same or uniform reimbursement and incentive mechanisms, announcing the pharmacy service activities for community on COVID-19 prevention

1. **Best practice**
2. What best practices does your institution have in ABHR production and utilization to prevent infections (COVID-19) epidemic?

Response**:** There is no best practice in our institutions.

1. Do you think your facility needs to be supported/strengthened/ with respect to ABHR solution production? If so, who do you think should support it? How do you think should it be supported?

*[Probe: training, material/resource]*

Response**:** Training (FMOH), Reference materials (Hospital), building for compounding (AARHB and Hospital), equipment support (FMOH and AARHB), continuous monitoring system (FMOH) and incentives (Hospital, FMOH, AARHB)

Comments: organogram for compounding services is mandatory and extra burden is a major problem for product interruption. Thus, assigning on one personnel is good to assure continuous production of ABHR.

**Participant #11**

| Age | 29 |
| --- | --- |
| Gender | Male |
| Highest qualification | B. Pharm |
| Current practices setting | General hospitals |
| Present position | Compounding case team head |
| Year of experiences, total(year) | 5 |
| Year of working experiences in the current position (years) | 3 months |
| When did your facility start production? | After COVID-19 Pandemic |

1. **Nature of formulation**
2. Do you follow World Health organization’s guideline for production of ABHR?

[*Probe: If “No”, ask formulation ingredients and amount to be used during production]*

Response: Yes, the procedures and type of ingredients for ABHR production are as per WHO guidelines.

1. **Availability and supply of chemicals, materials, PPE**
2. Is there problem in the supply of ingredients, personnel protective equipment, packaging and labelling materials? *[probe: reasons of unavailability: cost or procurement difficulties]*

Response: Yes, there is scarcity of ethyl alcohol and dispensing bottles. The main reasons are financial constraints, lengthy purchasing process, the is extensive demand as the facility is the major COVID center for the country, as the hospital is affiliated with St. Amanuel Mental Specialized Hospital.

1. What is the source of your active ingredient (ethanol or isopropyl alcohol), other chemicals and packaging and labelling materials? *[Probe: sugar industry, chemical industry, purchase from whole sale, obtained locally, imported, cost of 100 ml dispenser (for comparison)]*

Response: The main supply of raw materials like Glycerol and hydrogen peroxide is from PFSA but ethanol is obtained from MoH. The packaging materials specially 100 ml bottles are bought from private limited companies. The sources for the required active ingredients is variable from time to time

1. Do the required equipment specifically for the production of ABHRs purchased or donated or had them already?

Response: Big size storage containers (locally called “Roto”) were donated from Ethiopian Public Health Institute (EPHI). We got other facilities like tables from donors.

1. **Standard of practice**
2. Do you think that the production of ABHR solution at your facility comply the requirement or follow GCP principles? *[Probe: regular calibration and/or qualification, relevant quality control activities ABHR production (alcohol content determination, H_2_0_2_ titrimetry)]*

Response: As beginner, we striving to maintain the standards of GCP although it is not adequate. But we are not assuring the quality control of H2O2. The kits for H2O2 quality control are not available in the market.

1. How do you compare the ABHR solution prepared at your facility with those obtained from market? Do you have preference? Why? *(Probe: Differences in effectiveness, quality, cost (100 ml of facility produced and purchased from market)*

Response: The products produced in the public hospitals are much better than those available in the market.

1. If you think there is difference in any of the attributes, what are the major reasons?

*(Probe: manufacturing procedures, regulation, type of ingredients etc.)*

Response: The quality, appropriate labelling, and cost of the in-house sanitizers are the parameters. For example, at this time, 250 mL sanitizer is sold in 60 Birr in the market where as 500 mL is sold with this price in our facility.

1. Do you think the manufactured ABHR solution at your facility satisfy the demand of your organization? Explain the reasons for the response? *Probe: production capacity (daily/weekly/monthly), barriers for adequate production]*

Response: Yes, we are able to satisfy the demand of our organization although there is extensive demand from the patients and professional. We produce 100 litter per day.

1. Do your organization support/sell the product to other institutions/general public? *(Probe: agreement or contacts, the market demand)*

Response***:*** So far, we did not provide sanitizer for the public other than our hospital. But we have a plan to do so.

1. Does your organization have a plan to continue ABHR solution production after the COVID-19 pandemic is controlled globally?

Response***:*** Yes, we have the plan to continue the production of sanitizer and other dermatological agents after the elimination of COVID-19.

1. Do you estimate/assess the demand (daily or weekly) of ABHR solution at your faculty?

If yes, how? If no, why?

Response**:** Yes, we do have a daily consumption record. We breakdown the record for each of the departments in our hospitals.

1. **Rational use of ABHR**
2. Is there proper use of dispenser bottles by the health care professionals?

*(Probe: bring them properly for refill, request new if stolen or damage of pump or cup)*

Response**:** There is an appropriate utilization of the dispensing bottles. As this hospital is a COVID center we believe there is a better awareness and emphasis for the proper use of COVID related items. Still there are some losses of dispensing bottles.

1. Do you think there is rational use of ABHR solution at the facility? Mention reasons for your response?

Response**:** No, because ABHR is quantified and distributed by case team head. This type of distribution manners can easily wastage the products and cannot easily trace accountability.

1. Do you consider yourself to serve as highly accessible information expert and provide evidence-based information and education about sanitizer (ABHR)? If so, what type of information did you provide so far?

Response**:** Yes, nurses request information related with sanitizers. Types of information requested include, effect of the sanitizers, how to use, if it can be used to disinfect material like thermometer. Colleagues and neighbours also request information related to sanitizers.

1. **Professional**
2. Who is involved in production in your unit? *[Probe: pharmacists, nurses, chemists]*

Response: only pharmacists

1. Do they take training? If so, who is organize the training and when?

Response**:** No. formal training is not delivered for professionals but we took experiences from St. Amanuel Mental Specialized Hospital.

1. Do you believe that pharmacy professionals are duly recognized and reimbursed for their public health services to combat COVID-19 in Ethiopia? If not, why?

Response**:** No. I believe the pharmacist is fully engaged in the control of COVID-19 but they are not duly recognized and reimbursed for their public health services. For example, PPEs are not equally distributed for pharmacists as other health care providers. In this case N95 mask is not allowed for pharmacists, and supports from donors are neglected for pharmacists.

1. What supports are required to reimburse pharmacy professional for their service related with COVID-19 epidemic? *[Probe: accessing sanitizers, affordable price, advice in rational use, fair reimbursements]*

Response**:** supports for pharmacist should be fairly considers as other healthcare providers

1. **Best practice**
2. What best practices does your institution have in ABHR production and utilization to prevent infections (COVID-19) epidemic?

Response**:** Proper utilization of sanitizers through breakdown at department level.

1. Do you think your facility needs to be supported/strengthened/ with respect to ABHR solution production? If so, who do you think should support it? How do you think should it be supported?

*[Probe: training, material/resource]*

Response**:** MoH should arrange supportive supervision for facilities, EPHI, MoH and EPSA should provide supports in materials and equipment consistently.

Comments: we need consistent support from MoH.

**[Thank you!!!]**

**Participant #12**

| Age (years) | 30 |
| --- | --- |
| Gender | Male |
| Highest Qualification | MSc |
| Current practice setting | Specialized Hospital |
| Present position | Compounding coordinator |
| Year of experience, total (years) | 7 |
| Years of working experience in the current position (years) | 1 year |
| When did your facility start production? | Before COVID-19 Pandemic |

1. **Nature of formulation**
2. Do you follow World Health organization’s guideline for production of ABHR? [*Probe: If “No”, ask formulation ingredients and amount to be used during production]*

Response: Yes, it is based on formulation 1 (ethanol based)

1. **Availability and supply of chemicals, materials, PPE**
2. Is there problem in the supply of ingredients, personnel protective equipment, packaging and labelling materials? *[probe: reasons of unavailability: cost or procurement difficulties]*

Response: No: there is no as such problem of ingredients, PPE and packaging and labelling materials. Ethanol: at the starting time of manufacturing ABHR, there was problem of getting the required quantity but now it is solved as we are getting it through MOH facilitation. We need this facilitation to continue for future. Hydrogen peroxide and glycerol: they are easily available from market. Labelling and packaging: we are getting them easily from different suppliers (plastic factory and whole sale). PPE: no problem

1. What is the source of your active ingredient (ethanol or isopropyl alcohol), other chemicals and packaging and labeling materials? *[Probe: sugar industry, chemical industry, purchase from whole sale, obtained locally, imported, cost of 100 ml dispenser (for comparison)]*

Response: Ethanol: initially from EPSA but now from sugar industries (about 10000 liters sup to now) Hydrogen peroxide and glycerol: from EPSA and other whole sales. Labeling: from stationeries

Packaging: plastic factories and whole sales

1. Do the required equipment specifically for the production of ABHRs purchased or donated or had them already?

Response: most were purchased (e.g. measuring cylinder, jugs, beakers, alcoholmeter). We have also processing purchasing of additional equipment needed for production and mixing.

1. **Standard of practice**
2. Do you think that the production of ABHR solution at your facility comply the requirement or follow GCP principles? *[Probe: regular calibration and/or qualification, relevant quality control activities ABHR production (alcohol content determination, H_2_0_2_ titrimetry)]*

Response: our facility satisfied the basic requirements of GCP during production. Dedicated premise, available trained personnel, maintaining good personal hygiene and use of quality raw materials are important parameters for GCP. We also confirm the quality of compounded products by checking turbidity of solution, comprehensiveness of label information, alcohol concentration and others.

1. How do you compare the ABHR solution prepared at your facility with those obtained from market? Do you have preference? Why? *(Probe: Differences in effectiveness, quality, cost (100 ml of facility produced and purchased from market)*

Response: I recommend hospital-based ABHR than the commercial one as the health care facilities are not compounding ABHR for profit. Hospital-based ABHR fulfils all the quality requirements (e.g., 80% v/v of ethanol) compared to most commercial products. In addition, 130 ml ABHR solution is available for sale in our community pharmacy at a price of 30 Birr (less than $1 USD) against 100 Birr ($3 USD) for commercial product of same volume.

1. If you think there is difference in any of the attributes, what are the major reasons? *(Probe: manufacturing procedures, regulation, type of ingredients etc.)*

Response: Yes, the major reason is the quality of the products (e.g. ethanol strength). The poor control of commercial manufacturers by EFDA may also contribute for the difference.

1. **Production capacity and future plan**
2. Do you think the manufactured ABHR solution at your facility satisfy the demand of your organization? Explain the reasons for the response?

Probe: production capacity (daily/weekly/monthly), barriers for adequate production]

Response: Yes, we are producing beyond the demand of our facility and providing to other institutions as well. Currently we are preparing 200 liters per day though we have a capacity of 1000 liters per day.

1. Do your organization support/sell the product to other institutions/general public? *(Probe: agreement or contacts, the market demand)*

Response: Yes, we support different charity organizations by referring their simple official cooperation letter approved by our hospital CEO and pharmacy directorate. We have also sold our products through our community pharmacy as it is cost effective than the commercial product of private source.

1. Does your organization have a plan to continue ABHR solution production after the COVID-19 pandemic is controlled globally?

Response: Yes, it will be continued as it has been started before the pandemic.

1. Do you estimate/assess the demand (daily or weekly) of ABHR solution at your faculty? If yes, how? If no, why?

Response: Yes, we gave initially pocket-size dispenser (130 ml) and 500 ml for office purpose for a week and see whether there is complain or not. And we found it suitable for users.

1. **Rational use of ABHR**
2. Is there proper use of dispenser bottles by the health care professionals?

*(Probe: bring them properly for refill, request new if stolen or damage of pump or cup)*

Response: Yes, we have informed users first that such substitution of bottles (because of loss or damaged) will not be acceptable. Hence, there is no such issue raised before which might indicated the proper use of dispensers.

1. Do you think there is rational use of ABHR solution at the facility? Mention reasons for your response?

Response: Yes, there is considered as life savers. They are properly following the schedule for refill of the bottles or to take their weekly supply as per the estimated demand which indicates that they are using it rationally.

1. Do you consider yourself to serve as highly accessible information expert and provide evidence-based information and education about sanitizer (ABHR)? If so, what type of information did you provide so far?

Response: Yes, there we have disseminated brochures (when and how to use and its importance) in appropriate places of the hospital. additionally, we are informing when they appeared for refill.

1. **Professional**
2. Who is involved in production in your unit? *[Probe: pharmacists, nurses, chemists]*

Response: 6 pharmacists

1. Do they take training? If so, who is organize the training and when?

Response: Yes, one person took and others are taking his experience

1. Do you believe that pharmacy professionals are duly recognized and reimbursed for their public health services to combat COVID-19 in Ethiopia? If not, why?

Response: No. It is well known that COVID-19 is a serious health problem and the contribution of pharmacy professionals in saving life during this pandemic is significant. The necessary PPE and other resources for COVID-19 are mainly forecasted, purchased and distributed to other healthcare providers and patients by pharmacy professionals. But the attention given to the role of pharmacy professionals in this regard is not adequate (e.g., not considered for additional benefits like others healthcare providers) because they are not considered as front-line workers.

1. What supports are required to reimburse pharmacy professional for their service related with COVID-19 epidemic? *[Probe: accessing sanitizers, affordable price, advice in rational use, fair reimbursements]*

Response: pharmacy professionals should present their evidence of contribution in reasonable way and attention should be given. Officials from MoH and responsible organizations should clearly understand, recognize and acknowledge the role of pharmacy professionals during the pandemic.

1. **Best practice**
2. What best practices does your institution have in ABHR production and utilization to prevent infections (COVID-19) epidemic?

Response: We have separate compounding facility and dedicated team and responsible hospital management staff.

1. Do you think your facility needs to be supported/strengthened/ with respect to ABHR solution production? If so, who do you think should support it? How do you think should it be supported?

*[Probe: training, material/resource]*

Response: Yes, we need consistent supply of ethanol and follow up of the ABHR production in the hospital should be done by MOH and convince the hospital management as it is the standard of practice.

**If you have further points or comments to add, we will be appreciating.**

I want to emphasize that pharmacy professionals should get proper recognition from different organizations especially from MOH.

**[Thank you!!!]**

**Participant #13**

| Age (years) | 32 |
| --- | --- |
| Gender | Female |
| Highest Qualification | MSc |
| Current practice setting | Specialized Hospital |
| Present position | Pharmacy head and compounding coordinator |
| Year of experience, total (years) | 10 |
| Years of working experience in the current position (years) | 9 |
| When did your facility start production? | After COVID-19 Pandemic |

1. **Nature of formulation**
2. Do you follow World Health organization’s guideline for production of ABHR?

[*Probe: If “No”, ask formulation ingredients and amount to be used during production]*

Response: Yes, it is based on formulation 1 (ethanol based)

1. **Availability and supply of chemicals, materials, PPE**
2. Is there problem in the supply of ingredients, personnel protective equipment, packaging and labelling materials? *[probe: reasons of unavailability: cost or procurement difficulties]*

Response: No: there is no as such problem of ingredients, PPE and packaging and labelling materials. We have sufficient supply of all raw materials required for production. We have enough PPE obtained from mainly by donation and some from our stock. We got bottles (about 5000) for packaging purchased from local companies for the first time but we cannot get it in consistent way. The other is about labelling materials which we couldn’t get the right one (printed self-adhesive) rather than using the common printed paper to be fixed with glue.

1. What is the source of your active ingredient (ethanol or isopropyl alcohol), other chemicals and packaging and labelling materials? *[Probe: sugar industry, chemical industry, purchase from whole sale, obtained locally, imported, cost of 100 ml dispenser (for comparison)]*

Response: we get ethanol initially from national Sugar factory but nowadays the supply is from Fincha and/or Metehara sugar factory. We are getting glycerol and hydrogen peroxide from EPSA and other local suppliers. The DW is obtained from the hospital distiller.

packaging and labelling materials are purchased from the local suppliers

1. Do the required equipment specifically for the production of ABHRs purchased or donated or had them already?

Response: all are purchased specifically for this purpose like chemicals, equipment and mixing tanks mainly from EPSA.

1. **Standard of practice**
2. Do you think that the production of ABHR solution at your facility comply the requirement or follow GCP principles? *[Probe: regular calibration and/or qualification, relevant quality control activities ABHR production (alcohol content determination, H_2_0_2_ titrimetry)]*

Response: while we are preparing ABHR based on the WHO guideline, we are following GCP. We are using the required standard ingredients including distilled water which is obtained from calibrated distiller. We are also wearing all appropriate personal protective equipment (PPE) during production. We clean the compounding premise and production equipment before and after ABHR production regularly. We have documented all ABHR products distributed to the users.

1. How do you compare the ABHR solution prepared at your facility with those obtained from market? Do you have preference? Why? *(Probe: Differences in effectiveness, quality, cost (100 ml of facility produced and purchased from market)*

Response: Yes, there is difference, there is quality concern of some ABHR product from market which is manifested with longer time requirement (>30 sec) for drying on hand after application. This might be because of less alcohol strength. So, definitely there are quality and effectiveness differences with the in-house product superior.

1. If you think there is difference in any of the attributes, what are the major reasons? *(Probe: manufacturing procedures, regulation, type of ingredients etc.)*

Response: Yes, this is due to poor control over these products on market by EFDA. Due to this we have more products being sold in the market on streets like other goods over sunny.

1. **Production capacity and future plan**
2. Do you think the manufactured ABHR solution at your facility satisfy the demand of your organization? Explain the reasons for the response? Probe: production capacity (daily/weekly/monthly), barriers for adequate production]

Response: : sure, we are satisfying the facility demand. Through experience of previous delivery of about 100 ml twice a week, we know that this was not sufficient as further demand was raised. After that, we are providing about 1 liter for a period of 3 - 4 weeks, and this really satisfied the demand of our hospital. In addition to this personal use, we are providing ABHR for different wards to be used from wall mounted containers. Our production capacity is about 3000 liters/month.

1. Do your organization support/sell the product to other institutions/general public? *(Probe: agreement or contacts, the market demand)*

Response: Yes, we support different organizations by referring their simple official letter requesting cooperation. FMOH, EPSA, INSA, ministry of foreign affairs, Health facilities are among the others.

1. Does your organization have a plan to continue ABHR solution production after the COVID-19 pandemic is controlled globally?

Response: Definitely, as we have started production before COVID 19, it will continue through this pandemic will be under control. Because there are evidences of reduction of hospital acquired infections and other infectious diseases like cholera, GI related diseases, common cold etc

1. Do you estimate/assess the demand (daily or weekly) of ABHR solution at your faculty?

If yes, how? If no, why?

Response: Yes, it is based on rough estimation of ABHR consumption with the number of staff. This is done by checking the consumption data from the previous distribution record.

1. **Rational use of ABHR**
2. Is there proper use of dispenser bottles by the health care professionals?

*(Probe: bring them properly for refill, request new if stolen or damage of pump or cup)*

Response: No, we gave a 125 ml capacity bottles for all users at the beginning. But most of them came without them for the refill schedule and their reasons were loss of the bottle, broken caps etc. This shows the level of attention given to the bottles.

1. Do you think there is rational use of ABHR solution at the facility? Mention reasons for your response?

Response: No, there is an overall awareness problem among ABHR users in our facility. They don’t really know when and how ABHR is used. They are using ABHR repeatedly without touching patients or patient surroundings. There is also inappropriate volume of ABHR for a single use (more or less than 2 ml).

1. Do you consider yourself to serve as highly accessible information expert and provide evidence-based information and education about sanitizer (ABHR)? If so, what type of information did you provide so far?

Response: Yes. We, pharmacy professionals, have basic knowledge about rational use of medicines including sanitizers. In our facility**,** we provide as much information as we can via social media, leaflets and posters on notice boards which are prepared by members of the hospital drug information center. The information provided include what ABHR is, its ingredients, how to use it and precautions during its usage etc.

1. **Professional**
2. Who is involved in production in your unit? *[Probe: pharmacists, nurses, chemists]*

Response: pharmacists in rotation as the compounding period is once in week

1. Do they take training? If so, who is organize the training and when?

Response: Yes, one pharmacist took but we taught each other by reading guidelines

1. Do you believe that pharmacy professionals are duly recognized and reimbursed for their public health services to combat COVID-19 in Ethiopia? If not, why?

Response: No, we haven’t got due consideration from different parties (government, media, general public) regardless of great efforts from pharmacists (in terms of production, supplies availability, others). The reason may be because of lack of pharmacists’ approach to media,

1. What supports are required to reimburse pharmacy professional for their service related with COVID-19 epidemic? *[Probe: accessing sanitizers, affordable price, advice in rational use, fair reimbursements]*

Response: the media should invite pharmacists as well. We ourselves should make our role visible by others.

1. **Best practice**
2. What best practices does your institution have in ABHR production and utilization to prevent infections (COVID-19) epidemic?

Response: We have separate compounding premise which started ABHR production ahead of COVID 19 globally as IPC program. We have strong supply chain system which makes all necessary ingredients available on time. We also consistently produce and distribute to users without interruption.

1. Do you think your facility needs to be supported/strengthened/ with respect to ABHR solution production? If so, who do you think should support it? How do you think should it be supported?

*[Probe: training, material/resource]*

Response: We need a means of confirming the strength of hydrogen peroxide and glycerol raw materials. Supply of ethanol to healthcare facilities should be easy and continuous (available from nearby suppliers when required). Standard packaging bottles with appropriate sizes should be made available to us and this can be facilitated by MoH or other relevant stakeholders.

**If you have further points or comments to add, we will be appreciating.**

I want to thank MOH, PMED for consistently supporting our facility. I also thank CEO of our hospital and the pharmacy team for giving much attention and participating in ABHR production.

**[Thank you!!!]**
