# Supplementary material for: Perspectives of compounding pharmacists on alcohol-based hand sanitizer production and utilization for COVID-19 prevention in Addis Ababa, Ethiopia: A descriptive phenomenology study
Source: PLoS One. 2021 Apr 29;16(4):e0250020. doi: 10.1371/journal.pone.0250020 (PMC8084187; doi:10.1371/journal.pone.0250020)
Supplement: Attachment — Submitted filename: Birhanu_Reviewer Comments Nov , 2020.docx [file pone.0250020.s004.docx]

**General comments**

This is an interesting and informative manuscript looking closely about perspectives of compounding pharmacist on alcohol-based hand sanitizer production and utilization for COVID-19 prevention. The area is worthy of investigation; as relatively little exploration addresses this specific topic. Thus, this would be of interest to readers.

While navigating through the manuscript, there are some punctuation, capitalization and grammar errors that need to be corrected. And I would recommend the manuscript to be proofread again.

**Specific comments to each section of the manuscript**

**Abstract**

1. Would authors give explanation about the intent of using two different technical terms “**descriptive**” phenomenology study in the title of the manuscript and “**exploratory**” qualitative study in the abstract section?
2. Better to use “**in-depth interviews** of key-informants for phenomenological study”
3. It is better to use “**Findings**” instead of “Results” in the qualitative study.
4. It would be good to mention who are the **participants or the key informants** at least in the beginning of the paragraphs in the results and conclusion section of the abstract. Just like, “The compounding pharmacists in this study had a mean age of 30.6 (±3.1).”

**Introduction**

1. Every word should be in **non-abbreviated** form at its first appearance/ mention like COVID-19 and COREQ) (S2_File) etc.
2. Page 4, Line # 63, authors should delete “is” and rewrite it as “As ABHR has low irritation to hands….”
3. Page 4, Line # 81, authors justified the relevance of the study using evidences. One of the evidence is emergence of microorganisms resistance to the alcohol in hand sanitizers. Would authors support this with additional literatures?

**Methods**

1. Page 5, Line # 103: the type of sampling for the selection of participants in this qualitative study should be explained.
2. Page6, Line # 108 and 109, **Setting section:** It is appropriate to mention the total number of public hospitals in Addis Ababa in the setting part. “The recruitment of key informants was continued until the saturation point was reached at 13th interview and no new information was obtained from the subsequent interviews [17].” ….. this implies there are other possible interviewees.
3. Page 5, Line 111 : **Interview tool** …. Pls mention here about the use of conducting pilot test in the standardization of the interview tool.
4. **Reflexivity** –the positionality and perspectives of the researchers is missed in the manuscript. So it is good to incorporate it as it will affect or shape the whole research findings in quality study.
5. Page 7, Line # 138-142, so long as you use **thematic** **content** analysis, it is quite clear to follow and adhere to the steps in the thematic analysis. Do you feel it is important list down the steps here?

**Results …. Findings**

1. Page 9, Line 167, Table 1: Sociodemographic characteristics of the participants (N = 13). Authors should limit the number of vertical and horizontal lines as minimum as possible.
2. It is more advisable for qualitative researcher to have more narration of themes followed by peculiar quotes/ excerpts that underpin it.
3. On the other hand, the phrase “…**illustrated by the following excerpts**.” is recurrently appears in this section. So, better to reword it to enhance the palatability of the manuscript to the reader.

**Discussion and discussion**

It is well narrated section. However, I would recommend couple of points for authors.

1. The authors should argue strongly through comparing their findings with findings and experiences of other studies in other settings. So, the discussion should be anchored deeply in literatures.
2. The authors should distill recommendations for policy input and present them in the discussion well.
