# Supplementary material for: Perspectives of compounding pharmacists on alcohol-based hand sanitizer production and utilization for COVID-19 prevention in Addis Ababa, Ethiopia: A descriptive phenomenology study
Source: PLoS One. 2021 Apr 29;16(4):e0250020. doi: 10.1371/journal.pone.0250020 (PMC8084187; doi:10.1371/journal.pone.0250020)
Supplement: Attachment — Submitted filename: Response to Reviewers.docx [file pone.0250020.s005.docx]

**Point-by-point response**

| SN | Comments | Responses |
| --- | --- | --- |
|  | **Reviewer #1** |  |
| 1 | General comments |  |
| 1.1 | There are some punctuation, capitalization and grammar errors that need to be corrected. The manuscript to be proofread again. | The manuscript is proofread and punctuations, capitalization and grammar errors are corrected accordingly |
| 2 | Specific comments |  |
| 2.1 | Abstract |  |
| 1) | Would authors give explanation about the intent of using two different technical terms “descriptive” phenomenology study in the title of the manuscript and “exploratory” qualitative study in the abstract section? | To be consistent and appropriate according to the nature of the study, we managed it to be “descriptive” phenomenology study as indicated in the title. |
| 2) | Better to use “in-depth interviews of key-informants for phenomenological study” | Corrected accordingly in the abstract part of the manuscript |
| 3) | It is better to use “Findings” instead of “Results” in the qualitative study. | Yes, we replaced “Results” by “Findings” |
| 4) | It would be good to mention who are the participants or the key informants at least in the beginning of the paragraphs in the results and conclusion section of the abstract. Just like, “The compounding pharmacists in this study had a mean age of 30.6 (±3.1).” | Thank you, we put descriptions of the key informants as recommended (in the result and conclusion section of the abstract) |
| 2.2 | Introduction |  |
| 1) | Every word should be in non-abbreviated form at its first appearance/ mention like COVID-19 and COREQ) (S2_File) etc | As recommended from the reviewer, the non-abbreviated form of COVID-19 is provided as “coronavirus disease of 2019”. The long form of COREQ is also indicated in the manuscript as ”COnsolidated criteria for REporting Qualitative research” |
| 2) | Page 4, Line # 63, authors should delete “is” and rewrite it as “As ABHR has low irritation to hands….” | Thank you, “is” is deleted. |
| 3) | Page 4, Line # 81, authors justified the relevance of the study using evidences. One of the evidences is emergence of microorganisms, resistance to the alcohol in hand sanitizers. Would authors support this with additional literatures? | The reference indicated as reference #12 was changed to address the original article. An additional article on the efficacy of hand sanitizers is included as indicated in reference # 13. Therefore, the former reference numbers after ref #12 are relabeled. |
| 2.3 | Methods |  |
| 1) | Page 5, Line # 103: the type of sampling for the selection of participants in this qualitative study should be explained | Indicated as “purposive” |
| 2) | Page6, Line # 108 and 109, Setting section: It is appropriate to mention the total number of public hospitals in Addis Ababa in the setting part. “The recruitment of key informants was continued until the saturation point was reached at 13^th^ interview and no new information was obtained from the subsequent interviews [17].” ….. this implies there are other possible interviewees. | The total number of public hospitals in Addis Ababa is 13. In few of these hospitals the number of compounding pharmacists (pharmacy professionals) is beyond the total number of the hospitals. As we reached at the point of saturation of information, we ended to proceed the in-depth interview. If there were no saturation at that point, we could have additional number of interviews with in these hospitals |
| 3) | Page 5, Line 111: Interview tool …. Pls mention here about the use of conducting pilot test in the standardization of the interview tool | As indicated in the “study rigor” section of the manuscript, a pilot test was conducted at Adama General Hospital. The purpose of the pilot test is stated in the revised version of the manuscript. |
| 4) | Reflexivity –the positionality and perspectives of the researchers is missed in the manuscript. So, it is good to incorporate it as it will affect or shape the whole research findings in quality study | Reflexivity is incorporated in the revised version of the manuscript as” We tried to maintain reflexivity and avoid our own opinion from affecting the study data by precisely reviewing interview transcripts, comparing codes with the raw data, and checking the findings with the participants’ views several times”. |
| 5) | Page 7, Line # 138-142, so long as you use thematic content analysis, it is quite clear to follow and adhere to the steps in the thematic analysis. Do you feel it is important list down the steps here? | We are convinced to omit the steps that were listed in the manuscript as it is clear to follow and adhere to these steps. |
| 2.4 | Results (findings in the revised manuscript) |  |
| 1) | Page 9, Line 167, Table 1: Sociodemographic characteristics of the participants (N = 13). Authors should limit the number of vertical and horizontal lines as minimum as possible | The frequency and percentage of the study participants are made in a single column in the revised manuscript |
| 2) | It is more advisable for qualitative researcher to have more narration of themes followed by peculiar quotes/ excerpts that underpin it. | General introduction regarding the list and description of themes was already included. As much as possible, further narration of themes was included in the revised version of the manuscript |
| 3) | On the other hand, the phrase “…illustrated by the following excerpts.” is recurrently appears in this section. So, better to reword it to enhance the palatability of the manuscript to the reader. | Addressed accordingly in the revised version of the manuscript |
| 2.5 | Discussion |  |
| 1) | The authors should argue strongly through comparing their findings with findings and experiences of other studies in other settings. So, the discussion should be anchored deeply in literatures. | An additional literature is included. Publications are very limited on ABHR utilization and production. |
| 2) | The authors should distill recommendations for policy input and present them in the discussion well. | Recommendations for policy inputs are incorporated in the revised version of the manuscript |
|  | **Reviewer #2** |  |
| 1) | Two material sources were recommended to refer | We have considered one of the sources recommended for the discussion of our manuscript |
| 2) | The study was performed in 13 hospitals in the capital city. A very small sample of staff members took part in the project to make a larger impact | We appreciate your valuable comments. This is a qualitative research so that the number of participants is determined by the adequacy/ saturation of information gathered. |
| 3) | Additionally, you mentioned about the role of compounding pharmacists but it seems they had different education levels. Please explain, why there are different levels of education and mention this in the text please and if education may influence answers. Older pharmacist are much better train in compounding than young ones | The educational levels of the compounding pharmacists are either first degree or second degree. Any of them could be assigned by the hospital manager to coordinate the compounding pharmacy service usually after they are trained on the services. We have included the age of the study participants with the Quotes they provided. |
| 4) | Do you have an up to date numbers from Ethiopian Statistical Office? The 2007 population and housing census of Ethiopia is very old, and I am sure that many things has changed. What is an average number of produced sanitizers in all this 13 hospitals? Please calculate the volume. | Thank you. We made correction for this. In the revised manuscript, the projected population of Addis Ababa is 3.6 million in 2019. To calculate the production volume of each hospital was no the interest of the research, rather to explore if the hospitals were able to produce ABHR by their full capacity inline with their demand. |
| 5) | My final question, why did you decided to use this methodology, if this kind methodology is commonly used in nursing and midwifery research mainly? | The research question we raised demands this methodology. As the research questions are perspectives of pharmacists on the standards of ABHR production, barriers and challenges faced, perspectives on the rational use of ABHR, expectations … therefore, we believe that these types of questions are better addressed by qualitative methods than quantitative ones. The methods are so far utilized in pharmaceutical fields too‼ |
| 6) | I am missing the study limitation. I would not make my decision based on such a small number of participants, this was rather to show the problem, not to make an embracing change | The limitation of the study is already indicated in the final paragraph of the discussion in the manuscript. As stated above number of participants for qualitative studies is determined by the saturation level of information gathered |
|  | **Reviewer #3** |  |
| 1) | How do you evaluate the content validity of the interview guide? It has to be clearly described | Pilot testing of the tool we developed was employed as the method of content validation. This is stated in the manuscript (under the “study rigor”) |
| 2) | While the topic and objective of the manuscript is to explore the perspective and experience of compounding pharmacists on production and utilization of ABHR solution, the interview guide consists of question #15 and 16. For example, Participants were asked to reflect on their believe on how they are duly recognized and reimbursed for their public health services to combat COVID-19 in Ethiopia | These questions were included after the pilot test of the interview guide. The perspectives of compounding pharmacists on the compounding of ABHR was highly connected to their recognition and reimbursement for their activities. |
| 3) | How many pharmacies were there in Addis Ababa? how many of them were producing ABHR at the time of data collection? how do you select study participants (sampling technique if any) .....need to be clarified so that the readers may evaluate the representativeness of the sample used | As indicated in the method section of the manuscript, the study participants were those compounding pharmacists working at “public hospitals” not “pharmacies”. The selection of the study participants was purposive, those key-informants (compounding pharmacists). This is also indicated in the “setting, sampling and participants” part of the manuscript. |
| 4) | better to use most recent data on population of AA | Corrected, updated data is included in the revised version of the manuscript, 2019 projected population of Addis Ababa |
| 5) | …."The recruitment of key-informant was continued until the saturation point was reached at 13th interview and no new information was obtained from the subsequent interviews…." VS “ ….When no addition information was found from participants the content of the interviews was deemed to be saturated”......clarify the two different statements. What do we mean saturation point in qualitative study? | The later was indicated to explain the former sentence. In qualitative research, saturation is reached when no additional information was found from previous interviews. Sentence construction is revised in the manuscript. |
| 6) | The statement “On the reverse, few did not perceive that pharmacists are information experts regarding sanitizers use” does not support the excerpt which follows it…needs modification | Thank you very much, the statement is corrected as “On the reverse, few did not perceive that pharmacists are **not the only** information experts regarding sanitizers use which could be illustrated by the following excerpt…” |
| 7) | The language used while translating the Amharic tape record or note to English (excerpts) needs revision for grammatic errors | Revision is made in the revised version of the manuscript |
