# Supplementary material for: Perspectives of compounding pharmacists on alcohol-based hand sanitizer production and utilization for COVID-19 prevention in Addis Ababa, Ethiopia: A descriptive phenomenology study
Source: PLoS One. 2021 Apr 29;16(4):e0250020. doi: 10.1371/journal.pone.0250020 (PMC8084187; doi:10.1371/journal.pone.0250020)
Supplement: Attachment — Submitted filename: Response to Reviewers.docx [file pone.0250020.s006.docx]

**Point-by-point response**

| SN | Comments | Responses |
| --- | --- | --- |
|  | General |  |
| 1 | This article does not meet publication criteria #5 <https://journals.plos.org/plosone/s/criteria-for-publication>. PLOS ONE does not copyedit accepted manuscripts, so the language in submitted articles must be clear, correct, and unambiguous. I recommend that authors seek independent editorial help before submitting a revision | In this revision we made all possible correction to make the manuscript clear, and correct, and unambiguous. A senior professor who is expert in the field of the study was invited and made very important corrections. |
|  | **Abstract** |  |
| 2 | Page 2 Line 34: Add more details about the methods. For example, how were the pharmacists selected? | Further descriptions are included in this revision in the method section of the abstract. |
| 3 | Page 2 Line 36-37: “The compounding pharmacists in this study had a mean age of 30.6 (±3.1) years and men were the dominant one.” Instead, of “men were the dominant one,” you should provide the descriptive stats for what proportion were male | The descriptive stats for the proportion of men is indicated with the actual figure as “…mean age of 30.6 (±3.1) years and nine of the thirteen participants were men.” |
| 4 | Lines 37-38: “Most participants believed that the compounding practice with their respective sites followed the principles of good compounding practice.” Define what “most participants” means. Provide the data. | It is correcting by specifying the actual number of participants that maintain the principle of good compounding practice. It is rewritten as: “Ten participants believed that the compounding practice in their respective sites followed the principles of good compounding practice.” This is also corrected in the main result section of the manuscript. |
| 5 | Lines 41-43: “Study participants demand incentive mechanisms and reimbursements for experts involved in compounding of ABHR solutions.” Did they “demand” incentives or is there a better word choice like “suggested” or “recommended.” | Thank you for the “soft words” suggested. We modified the sentence as “Study participants **suggested** incentive mechanisms and reimbursements for experts involved in **the** compounding of ABHR solutions.” |
| 6 | Lines 45-47: “Few of participants the compounding pharmacists indicated that the compounding practice lack compliance of good compounding practice due to lack of convenient compounding room, quality control tests, sufficient manpower and equipment.” This is an awkward sentence that needs significant revision. What does “few of the participants” mean? The word “lack” is used twice which confuses the meaning in the sentence | The exact number (i.e., three) replaced “few” and the whole sentence is reconstructed as follows: “Three of the compounding pharmacists indicated that ABHR production in their setting lack compliance to good compounding practice due to inadequate compounding room, quality control tests, manpower and equipment.” |
|  | **Methods** |  |
| 7 | Page 6 Lines 110-111: “The compounding pharmacists who had agreed to participate in the study were approached.” Approached by whom? In what way were they approached? This sentence either needs to be clarified or deleted | They were approached by the research team. Revision is made in this way “Compounding pharmacists who had agreed to participate in the study were approached by the research team.“ Participants were approached after the objectives and the process of the study was explained to them, as stated in the data collection section of the manuscript. |
| 8 | Lines 111-113: “The recruitment of key- informants was continued until the saturation point was reached at 13th interview and as no new information was obtained from the subsequent interviews.” 13th should be spelled out as thirteenth. The word “the” should proceed 13th. This sentence is unclear as it suggests that 13 interviews were conducted but infers that more were conducted but that the subsequent interviews did not yield new information. Therefore, 13 interviews were used for the analysis but how many interviews were conducted? | The word “the” is included before 13^th^ (spelled out as thirteenth). We did thirteen interviews and we got the information collected at this point to be nearly similar to the preceding data. Therefore, we ceased further data collection. We have included all the thirteen interviews in the analysis. The way we stated this sentence is modified as follows: ”**The key-informant interview was continued until the thirteenth interview as the information gathered was saturated at this point.”** |
| 9 | Other corrections… this is not an exhaustive list, but illustrates the need for greater attention to the writing quality. | Corrections are made throughout the manuscript to address the clarity of the manuscript. |
